# Fungal Proteases as Emerging Biocatalysts to Meet the Current Challenges and Recent Developments in Biomedical Therapies: An Updated Review

**DOI:** 10.3390/jof8020109

**Published:** 2022-01-24

**Authors:** Muhammad Naeem, Saba Manzoor, Mashhud-Ul-Hasan Abid, Muhammad Burhan Khan Tareen, Mirza Asad, Sajida Mushtaq, Nazia Ehsan, Dua Amna, Baojun Xu, Abu Hazafa

**Affiliations:** 1 College of Life Science, Hebei Normal University, Shijiazhuang 050025, China; naeemsaleem413@gmail.com; 2 Department of Zoology, University of Sialkot, Sialkot 51310, Pakistan; saba.manzoor@uskt.edu.pk; 3 Department of Biochemistry, Bahauddin Zakariya University, Multan 60800, Pakistan; mashhoodabid@gmail.com; 4 College of Food Science & Technology, Huazhong Agricultural University, Wuhan 430070, China; burhan.tareen786@yahoo.com; 5 Department of Biochemistry, University of Agriculture Faisalabad, Faisalabad 38040, Pakistan; asadmirza074@gmail.com; 6 Department of Zoology, Government College Women University, Sialkot 51040, Pakistan; sajida.mushtaq@gcwus.edu.pk; 7 Department of Zoology, Wildlife and Fisheries, University of Agriculture Faisalabad, Faisalabad 38040, Pakistan; naziaeuaf@gmail.com; 8 Institute of Food Science & Nutrition, Bahauddin Zakariya University, Multan 60800, Pakistan; fatimadua11233@gmail.com; 9 Food Science and Technology Program, Beijing Normal University-Hong Kong Baptist University (BNU-HKBU) United International College, Zhuhai 519087, China

**Keywords:** fungal enzymes, proteases, catalytical properties, recent developments, genetic engineering, mitoproteases

## Abstract

With the increasing world population, demand for industrialization has also increased to fulfill humans’ living standards. Fungi are considered a source of essential constituents to produce the biocatalytic enzymes, including amylases, proteases, lipases, and cellulases that contain broad-spectrum industrial and emerging applications. The present review discussed the origin, nature, mechanism of action, emerging aspects of genetic engineering for designing novel proteases, genome editing of fungal strains through CRISPR technology, present challenges and future recommendations of fungal proteases. The emerging evidence revealed that fungal proteases show a protective role to many environmental exposures and discovered that an imbalance of protease inhibitors and proteases in the epithelial barriers leads to the protection of chronic eosinophilic airway inflammation. Moreover, mitoproteases recently were found to execute intense proteolytic processes that are crucial for mitochondrial integrity and homeostasis function, including mitochondrial biogenesis, protein synthesis, and apoptosis. The emerging evidence revealed that CRISPR/Cas9 technology had been successfully developed in various filamentous fungi and higher fungi for editing of specific genes. In addition to medical importance, fungal proteases are extensively used in different industries such as foods to prepare butter, fruits, juices, and cheese, and to increase their shelf life. It is concluded that hydrolysis of proteins in industries is one of the most significant applications of fungal enzymes that led to massive usage of proteomics.

## 1. Introduction

Excessive use of chemicals in different industries has increased tremendously in the past few years, including phenols, pesticides, polyaromatic hydrocarbons (PAHs), polychlorinated compounds, polychlorinated biphenyls, and arsenic. Exposure to these toxic chemicals has become a threat to the environment and public health concerns [1]. There is a need to replace these chemical compounds with fungal enzymes that are considered alternatives to the toxic chemicals and act as ecofriendly indicators to meet industrial demands [1]. The global market of fungal enzymes is drastically increasing in different sectors due to the high production rate, smooth downstream processing, and low costs [2,3].

Fungi are considered GRAS (Generally Regarded as Safe) organisms as compared to other microorganisms because they fulfill the criteria of industrial demands such as efficient growth on culture media in short duration and continuous supply of desired products [4,5]. Fungi also secreted a large variety of proteases, lipases, amylases, and amylases that play an important role in physiological processes such as germination, as defensins against other pathogens or for nutritional requirements for development [6,7]. Secretions of fungal enzymes occur from the cells present at the top of hyphae. These secreted enzymes can be used for industrial preparations of valuable products [8].

Fungal proteases have been widely studied due to their wide diversity [9]. Proteases have been isolated from different fungi such as *Schizophyllum commune*, *Pleurotus ostreatus*, *Phanerochaete chrysosporium*, *Thermomyces lanuginosus*, *Sporotrichum thermophiles*, *Myceliophthora thermophile*, *Thermomyces ibadanensis*, *Candida mogii*, *Saccharomyces pombe*, *Aspergillus flavus*, and *Neurospora crassa* [10,11,12]. Fungal proteases can be isolated through the fermentation process exhibiting high catalytic and specificity for the substrate [1,13]. 

Fungal proteases have diverse importance in corporate sectors such as the pharmaceutical, detergent, leather, waste, and food industries [14]. In the food industry, they are used to make beer, wine, and vinegar. Acidic proteases are used to improve wheat gluten’s structural and functional properties [15]. In medical sectors, fungal proteases are used as therapeutic agents for the treatment of a variety of diseases such as cancer, HIV, inflammatory diseases, diabetes, and hepatic cancer [16,17]. In the textile and laundry industries, they are used to prepare enzyme-based detergents to remove the tough stains from clothes due to developing excellent washing performance compared to other microbial enzymes [13,18]. They are also involved in degrading lignocellulosic biomass, and products can be utilized as biofuels for the production of energy at the commercial level [8,19].

Fungal proteases hold a pivotal position in cellular signaling and physiological processes occurring in the human body [20]. They possess antiviral, anti-inflammatory, antioxidant, and antitumor activities due to the presence of bioactive compounds [21]. Through recombinant DNA technology, different genes from fungal proteases have been cloned and sequenced in order to enhance their production under optimum conditions [22,23]. For instance, a selected gene that encodes target enzyme shows its expression in the fungal cell factory, such as *T. reesei*, *A. nidulans and A. oryzae*, *A. oryzae* FG76, *Pseudomonas aeruginosa* CTM50182 and *P. chrysogenum* (Pg222) [24,25,26,27,28]. The accumulative data also revealed that CRISPR/Cas9 technology had been successfully developed in different fungi, including *Trichoderma reesei*, *Aspergillus stains*, *A. fumigatus*, and *Ustilago maydis* for genome editing [29,30,31]. 

Many fungal strains have been isolated, but their diversity remains unexplored and a barrier for cost production prior to use for different industrial operations. Due to vast biodiversity, many fungal strains have been reported that secreted proteases, but their nature, secretary pathways, molecular structures in 3D, mechanism of action, and roles in cellular and physiological processes remain unclear. The impact of COVID-19 on the global market affecting fungal proteases, genetic expression, and genome editing of many strains through CRISPR technology needs to be explored before using them in different sectors.

However, this review highlights the origin, nature, mechanism of action, emerging aspects of genetic engineering for designing novel proteases, genetic expression and genome editing of fungal strains through CRISPR technology, and roles in cellular and physiological processes. This review also presents the impact of COVID-19 on global market trends affecting fungal proteases. 

## 2. Fungal Enzymes

Different enzymes are isolated from different fungus strains and are used as detergents for softening and washing clothes to maintain their quality, color, and other cloth properties. These different fungal enzymes are proteases, amylases, cellulases, and lipases used as detergents to increase their efficiency [32,33,34,35]. The characteristics of different fungal enzymes are presented in Table 1. These enzyme-based detergents also have advantages over synthetic chemicals because they are biodegradable, environment-friendly, and are used in a minimal amount to remove strains [36].

### Coproduction of Fungal Enzymes

Fungi secrete different enzymes and peptides that play a significant role in cellular processes, growth, and sporulation. Fungi are classified as psychrophilic, mesophilic, and thermophilic on the basis of their particular habitat as these fungi produce a large variety of enzymes [77,78]. Some species of thermophilic fungi can tolerate high temperature and secrete a variety of proteases. Due to their longer shelf life, these proteases can be used in different industries, including food and pharmaceuticals [79]. Some fungi found in deep sea are also a major source of psychrophilic protease [80]. Different species of mesophilic fungi also contribute to the large-scale production of proteases [22]. *Thermomyces lanuginosus* shows optimum growth at 40–50 °C, and produces proteases, lipases, and amylases used to treat wastewater and the pharmaceutical industries [81,82]. *Neurospora crassa* shows optimum growth at 20–30 °C and produces alkaline, serine proteases, and cellulases used as a model organism to analyze genetic recombination [83].

Similarly, *Myceliophthora thermophile* shows optimum growth at 45–50 °C, produces alkaline protease and cellulases used for textile industries and bioremediation [84]. *Thermomyces ibadanensis* shows optimum growth at 55 °C, produces serine proteases and lipases used for wastewater treatment [85]. The detailed information about the coproduction of fungal enzymes from different fungal strains with their applications is presented in Table 2. 

## 3. Fungal Proteases

Proteases are obtained from plants, animals, and microorganisms. Plants and animals’ proteases are more complex as compared to microorganisms. Fungal proteases are used as detergents for the removal of stains by hydrolyzing the peptide bond among the protein molecules. Different strains of fungus produce the proteases, including *Aspergillus niger* [90]. Proteases have advantages over other fungus enzymes as detergents. Their demand increased due to low compatible detergent due to washing performance as compared to other enzymes. They catalyzed the removal of protein strains by breaking the peptide bonds in all pH ranges, such as acidic, neutral, and alkaline [91]. Their use in industrial and households increased due to the high production rate, enzyme recovery from the respective media, environmental safety, the whiteness of clothes, and maintenance of fibers.

Among these enzymes, protease is mainly used in detergent formulations due to its immense use in different industries. Proteases are referred to as proteolytic enzymes, which are unique in nature due to their presence in all living organisms and their significant applications in cell growth and differentiation. The proteases may be divided into three main groups based on the pH range alkaline protease, acidic protease, and neutral protease. Acidic protease has an optimum pH between 2.0 to 5.0. The ideal pH condition of neutral protease is 7.0, and alkaline protease has an optimum pH of more than 7.0. The acidic protease mainly has a fungus origin. Alkaline proteases are most important due to their immense applications in the food and detergent industries [92].

**Figure 1:**
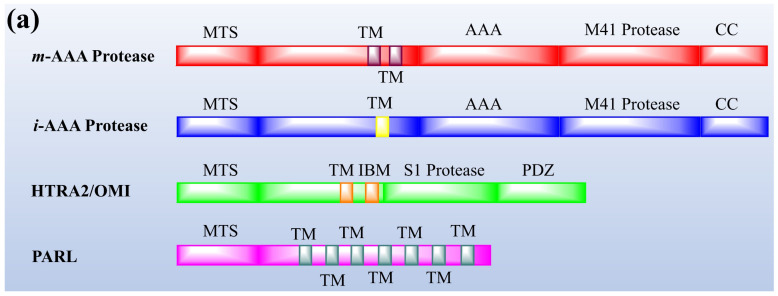

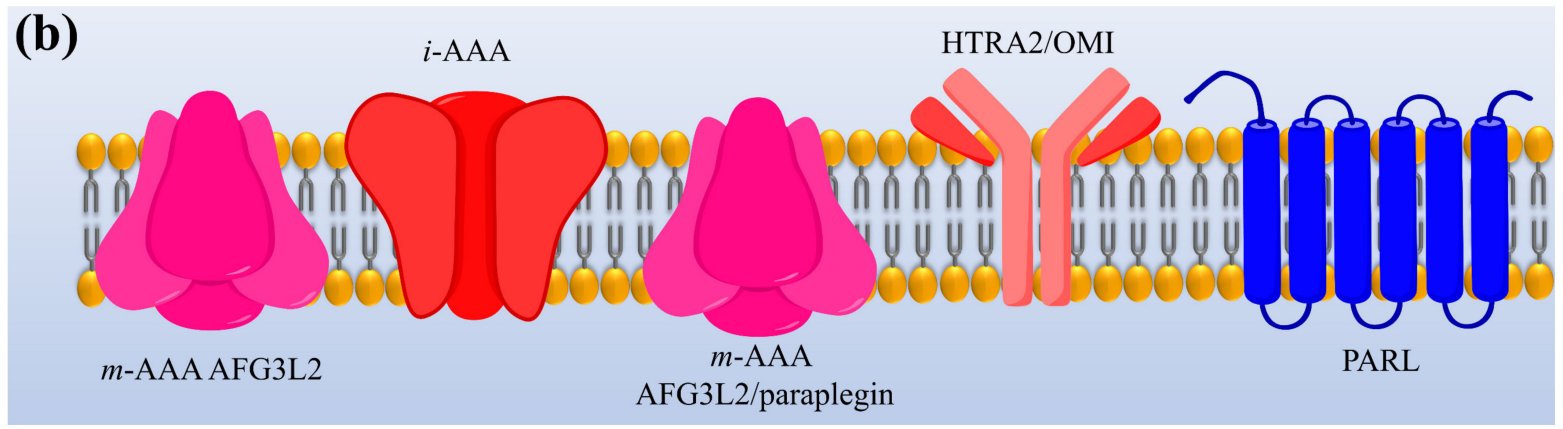
The representation of (**a**) structure and (**b**) topology of proteases. TM: Transmembrane domain, IBM: Inhibitor of apoptosis (IAP)-binding motif, MTS: Mitochondrial targeting sequencing, IMS: Intermembrane space, CC: Coiled-coil, AAA: Triple-A domain, M41: Protease metal-binding proteolytic domain, S1 protease: Trypsin-like protease domain. This figure is reproduced from Martinelli and Rugarli [94] after permission from Elsevier (License No. 5197711293142).

Protease enzymes are classified into two types based on their mechanism of action: extracellular protease and intracellular protease. The extracellular enzyme catalyses the hydrolysis of large protein molecules into smaller molecules that are absorbed into the cells. The intracellular protease plays a significant role in the regulation of metabolism. The extracellular protease enzyme is mostly used at a commercial scale to degrade protein molecules in many industrial processes [93]. The structures of different proteases are presented in Figure 1.

Alkaline proteases are used as detergents in the laundry industry to remove the dirty stains that are portentous in nature, in many food industries such as cheese making, baking meat industries, soaking processes, and many others in waste management from many food industries and also have some application in household activities [95]. Alkaline proteases act as an active ingredient in laundry detergent; they are considered a significant application of this enzyme. In the past, when detergent protease was produced at an industrial scale, it caused some allergic problems in workers from the dust of enzymes [96]. 

Among all groups or classes of proteases, the serine proteases are most effective in detergent action due to their stability and comparability in the existence of ingredients and other bleaching agents. It is expected that in 2020, the global market of industrial reach will climb to 7.5 billion and the growth rate will be around 8.2%. It is estimated that the growth rate will be maximum in detergents’ enzymes. One type of such enzyme, protease, remained a well-known enzyme of 2015; its growth rate was 27.5%. It is expected to enhance its growth rate because of its flexibility in different sectors of pharmaceutical foods and detergents [97]. The information about novel protease enzymes isolated from different sources is presented in Table 3.

### 3.1. Origin of Fungal Proteases

The demand for fungal proteases has increased in recent decades. Different species of fungi secrete proteases, but the origin of proteases from basidiomycetes remains unclear [110]. *Aspergillus* species are considered an excellent source of proteases. Some other fungal species such as Penicillium and Rhizopus also produce proteases [111,112]. Proteases are also produced from the basidiomycetes such as *Schizophyllum commune*, *Armillariella mellea*, *Pleurotus ostreatus*, and *Phanerochaete chrysosporium* [113,114,115].

Mycelial secretion in saprophytic basidiomycetes led to discovery of proteases such as subtilases from *Serpula lacrymans*, *Pleurotus ostreatus*, and *Irpex lacteus* [116,117]. *Pleurotus* sp., such as *P. ostreatus* and *P. chrysosporium*, also produced the proteases involved in ligninolytic mechanism through fragmented degradation of laccase enzyme during fugal growth [118]. Hemolytic proteases secreted by *Pleurotus* sp. possess different activities in cellular processes. Secretion of hemolysin by *P. nebrodensis* shows apoptosis and antiproliferative activities and is involved in targeting cancerous cells [119]. These proteases tightly bind to the receptor proteins of HIV and inhibit them [120]. Some proteases isolated from *P. ostreatus* possess activities against different carcinomas [121].

### 3.2. Classification of Fungal Proteases

Fungal proteases are classified into different categories based on amino acids. A few are presented in the following sections.

#### 3.2.1. Protease Classes Based on Amino Acids

Proteases are categorized into the following classes based on amino acids’ residue in their active site.

#### Serine Protease

**Figure 2:**
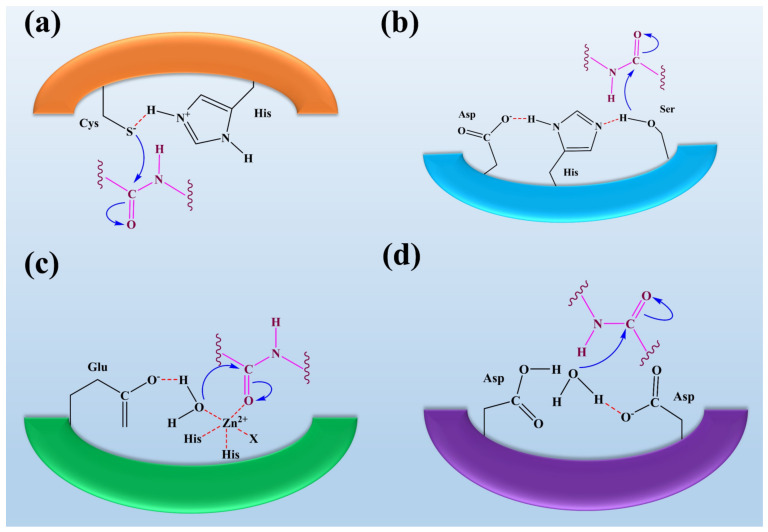
The representation of fungal protease mechanisms of (**a**) serine proteases (**b**) aspartyl proteases, (**c**) metalloproteases, and (**d**) cysteine proteases. It was reported that the eponymous residue is commonly formed as a pair with a proton withdrawing group in the active sites of cysteine and serine proteases to promote a nucleophilic attack on the peptide bond. In contrast, metalloproteases and aspartyl proteases activate water molecules as nucleophiles. Overall, it was observed that the process of peptide bond scission is the same for all classes of proteases. This figure is reproduced from Erez et al. [135] after permission from Springer Nature (License No. 5223461087907).

Serine proteases are the most important class of proteases that contain the amino acid serine in their active site (see Figure 2). Serine residues in the active site make a catalytic triad with aspartate and histidine. This catalytic triad is conserved among all serine proteases. These residues are essential for their catalytic activity to cleave the substrate protein [122]. Serine proteases can be further divided based on substrate specificities such as elastase-like proteases, which possess the smaller S1 cleft than the chymotrypsin-like protease. They are hydrophobic in nature and possess the specificity for valine and glycine. These elastase-like proteases act on elastin by breaking them into smaller fragments, thus playing an essential role in forming connective tissues [3]. Fungal species such as *Aspergillus* sp., *Aspergillus oryzae*, *Aspergillus fischeri*, *Penicillium citrinum*, *Penicillium corylophilum*. *Penicillium waksmanii*, and *Neurospora Conidiobol* produce serine proteases [123,124].

#### Threonine Protease

Threonine proteases hold the threonine residues in their active site for their catalytic activity to cleave the substrate protein. These proteases show substrate specificity for bulky amino acids [109,125]. *Saccharomyces cerevisiae* are production sources of threonine peptidase with appreciable capacity for the production of threonine and potential for industrial application [123,126].

#### Cysteine Protease

Cysteine proteases hold the two amino residues in their active sites, such as cysteine and histidine (see Figure 2). Their catalytic activity can be maintained in the presence of reducing agents [109]. The substrate specificity of the cysteine proteases can be determined through the interaction between the side-chain amino acids of the particular substrate that can be accommodated into the S2 cleft, which is hydrophobic in nature and shows the specificity for the leucine and tyrosine. One of the best examples is the Cathepsin K that shows the specificity for kinins by cleaving the peptide bonds in the collagen tissue [98,127]. Fungi species such as *Aspergillus oryzae* produce cysteine proteases. Only a little information about the secretion of fungal cysteine proteases is known [128].

#### Aspartate Protease

Aspartate proteases hold the two aspartate residues in their active site. These aspartate residues are essential for their catalytic activity to cleave the substrate protein (see Figure 2). Aspartate proteases show specificity for aromatics such as phenylalanine, tyrosine, and tryptophan on both sides of the peptide bond [101]. Rennin-like aspartate proteases cause the cleavage of casein into the smaller peptides. Pepsins are isolated from *Aspergillus*, and rennet-like enzymes are isolated from *M. miehei* [100]. Fungi species such as Aspergillus, Penicillium, Rhizopus, and Neurospora produce aspartic proteases [100,129]. *E. parasitica* and *R. miehei* of fungi are used as production sources of aspartic peptidase with appreciable capacity for the production of acidic peptidase and potential for industrial application [129,130].

#### Glutamic Acid Protease

Glutamic acid protease holds the glutamate residues in their active site. These proteases also show substrate specificity for bulky amino acids. For instance, eqolisin can work at pH 2.0 when the casein is used as a substrate. Glutamic proteases were identified in the fungi *Scytalidium lignicola* and *Aspergillus niger* [106,107]. *Scytalidium lignicolum*, *Aspergillus niger*, *Cryphonectria parasitica*, *Talaromyces emersonii* and *Sclerotina sclerotiorum* secreted glutamic proteases [131,132,133]. 

#### Metalloprotease

Metalloproteases are the diverse classes of proteases containing metal ions in their active sites (see Figure 2). These metalloproteases are highly specific in their action [104]. Neutral proteases show the specificity for hydrophobic amino acids. The well-known metalloproteases such as collagenases and elastase are isolated from *C. histolyticum* and *P. aeruginosa* [105]. Fungi species such as Aspergillus, Penicillium, *Fusarium oxysporum*, and *A. fumigatus* produce metalloproteases [100,126,134].

**Figure 3:**
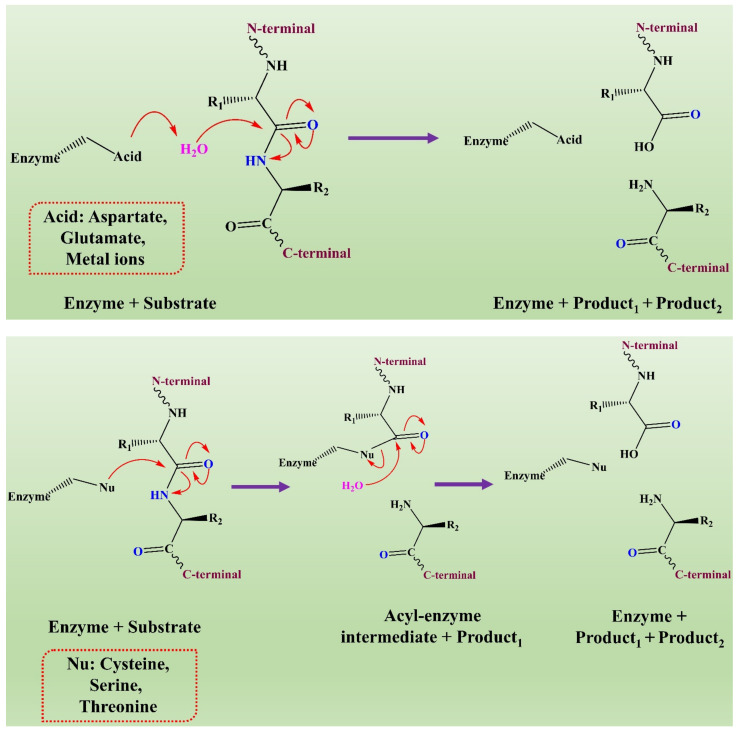
The representation of a comparison of the two hydrolytic mechanisms used for proteolysis. This figure is reproduced from Shafee [136] (Attribution NonCommercial 2.0 UK: England & Wales, CC BY-NY 2.0 UK).

The mechanism of action of fungal proteases involves the formation of an intermediate acyl-enzyme that covalently links the enzyme to the N-terminal of the substrate. In the second step, the water molecule completes the hydrolysis by attacking the intermediate. It results in the release of the second-half product with the regeneration of free enzyme (see Figure 3) [136].

## 4. Market Value of Fungal Proteases

Fungal proteases are one of the largest groups of industrial enzymes, and their global market is drastically increasing annually. Of the 60% of enzymes marketed worldwide, fugal proteases account for 20% [137,138]. The global market of fungal proteases is gaining more attention compared to other enzymes due to their high demand, catalytic properties, and low cost [139,140]. The global fungal enzymes market is highly consolidated owing to the presence of several key players operating in the global fungal enzymes market, namely Novozymes A/S, DSM, Chr. Hansen, DuPont, and BASF. The share market is divided into pharmaceuticals, bakery, beverages, sweet, and animal feed based on application [141]. Companies worldwide adopt evolutionary acquisition strategies by expanding the business network of proteases by considering the geographical demands, source type (especially microbial), and role in different industries [140].

### Global Proteases Market Segmentation

The global protease market is segmented based on applications, forms, and types. The market is divided into renin, trypsin, pepsin, and others based on type. The renin segment leads the share market, and alkaline protease is the second segment that accounts for the largest market share due to usage in the food and dairy industry [142]. Based on form, the protease market has been divided into liquid and powder. The powder segments contribute to the largest market share due to the usage of many products that improve the half-life of food products [90]. The share market is divided into bakery and beverages, sweet, animal feed, and others based on application. The bakery segment contributes to the largest share of the market. It reflects the use of proteases in different industries for syntheses of food-based products [143]. 

## 5. Molecular Approaches, Cloning and Expression of Fungal Proteases

Recombinant DNA technology is used for fungal proteases to understand their gene expression at cellular and molecular levels [144]. Protein engineering of fungal proteases can be evaluated through directed mutagenesis for introducing the changes in the sequence of amino acid 3D structure [22]. Based on a particular sequence at the N-terminal, novel proteases were isolated from *P. ostreatus*. A new primer was designed for cloning and amplification of DNA sequence that showed regions with homology to the proteases isolated from *Neurospora crassa* [111]. *XPR2* gene encoding for an alkaline protease from *Yarrowia lipolytica* was cloned into *P. pastoris*, and its genomic sequence showed two glycosylation sites [145]. A serine protease gene (*Spr1*) encoding from an alkaline protease from *Monacrosporium megalosporum* showed homology to serine proteases PII and Azo1 isolated from the cuticle of nematode-trapping fungus and *Arthrobotrys oligospora* [146]. Gene cloning of *A. nidulans* gene (*prtA*) encoding the alkaline protease can be carried out in the presence of probing of the *A. nidulans* library through fragmented amplification of the *Aspergillus oryzae Alp*-encoding gene. The amino acid sequence in the *prtA* gene showed homology to the protease isolated for *A. oryzae* and *A. flavus* [147,148]. 

Recently, a novel neutral protease was produced from *Aspergillus oryzae Y1*. This protease was purified about 10-folds, and its recovery yield was about 45% [149]. This protease was purified as a potential candidate for industrial applications, especially in food industries [149,150]. The genetic recombination of the *A. oryzae* and *Aspergillus* led to the production of *A. oryzae* FG76, which secreted acid protease with 85% activity [28]. Novel acid protease was produced from *A. oryzae* FG76 from a culturing medium with 17-activity and showed high stability as compared to commercial enzymes [28]. Another novel protease was purified from basidiomycete fungus CTM10057 that acted as a potential candidate for industrial applications, especially in the detergent and laundry industries [150,151]. *Pseudomonas aeruginosa* CTM50182 secreted the AMPP protease with 70% activity that showed high stability as compared to commercial enzymes [28,152]. *Cryptococcus* sp. S-2 that produced the novel aspartic protease was purified in synthetic substrates similar to the purified pig pepsin A [153].

## 6. Role of CRISPR Technology in Fungal Enzymes

CRISPR/Cas9 technology has been successfully developed in fungi for genome editing such as *Trichoderma reesei*, *Aspergillus stains*, *A. fumigatus*, and *Ustilago maydis* (see Table 4) [29,30,31]. For example, genome editing of multiple genes was successfully achieved in *T. reesei* through CRISPR/Cas9 technology by following the co-transformation via in vitro synthesis of sgRNAs and donor DNA with the 200 bp homologous arms. This promising approach applies to filamentous fungi that degrade the wood lignocelluloses [154]. The CRISPR/Cas9 system also successfully developed in *A. fumigatus* following in vivo synthesis of sgRNA through the introduction of *A. fumigatus* U6 snRNA promoter, leading to 95–100% precise integration via the 35-bp homologous arms. This versatile genome editing approach is helpful for the investigation of mutated genes and clinical studies of *Aspergillus* species [155]. Genome editing through CRISPR/Cas9 technology is under validation for thermophilic fungi [156]. *Myceliophthora thermophile* is one of the thermophilic fungi involved in the degrading of biomass; therefore, it can be used for the production of thermostable enzymes as biofuels in different industries. Consequently, it is essential to reveal the genome editing of Myceliophthora through metabolic engineering important in reconstructing the lignocelluloses [157]. 

However, the off-target effects in fungi have been studied through CRISPR/Cas9 technology. Mutations were observed in *U. maydis* and *A. fumigatus* through whole-genome sequencing after CRISPR/Cas9 genome editing [170,171]. There is a need to elucidate the mechanism behind mutations/off-target events through CRISPR/Cas genome editing in fungi [172]. Some strategies have been developed in order to reduce off-target effects caused by CRISPR/Cas-9 genome editing in fungi. Based on genomic sequence, algorithms can be used for the selection of target sequences for predicting the off-target and on-targets sites [173]. In fungi, joining non-homologous regions can be carried out via a null mutant strain to reduce off-target effects [174]. High homologous recombination was observed through deletion of genes that particularly involved non-homologous end-joining. Therefore, mutants have been used in the form of hosts to process CRISPR genomic editing [175].

## 7. Recent Developments

In this modern era, biocatalysts are considered important progress in environmental protection. The production of an industrially important enzyme by using a cheap carbon source involves the continuous isolation of new strains [176]. Protease activity depends on many factors, including the pH of the medium, mechanical handling, and temperature. Genetic engineering plays an essential role in producing novel enzymes with unique properties and alteration of protease properties that can improve the industrial process [139]. Proteases can overcome the traditional methods of biomass conversion into biofuels. Enzymes are the best alternatives for methyl ter-butyl ether (MTBE) because MTBE is dangerous to human health. Proteases have the potential to use unrefined feedstock without the separation of fatty acids that are mainly present in unrefined feedstock [90]. The complete genome of many industrial important *Aspergillus* species that produce various enzymes has been sequenced [177]. Despite limitations at transcriptional and translation sites, with the help of enzyme engineering, the *Aspergillus* species has the potential to produce a high level of heterologous proteins [15]. 

## 8. Role of Fungal Proteases in Living Organisms

Mitoproteases process the proteins imported into mitochondria and help degrade the damaged and misfolded proteins to improve the quality of proteins [178]. However, with the help of recent technologies, a mutation in genes that encodes mitoproteases and results in different diseases can be found, providing additional information regarding the biological role of these proteases in other processes [179]. 

### 8.1. Mitoproteases Function in Protein Processing and Activation

**Figure 4:**
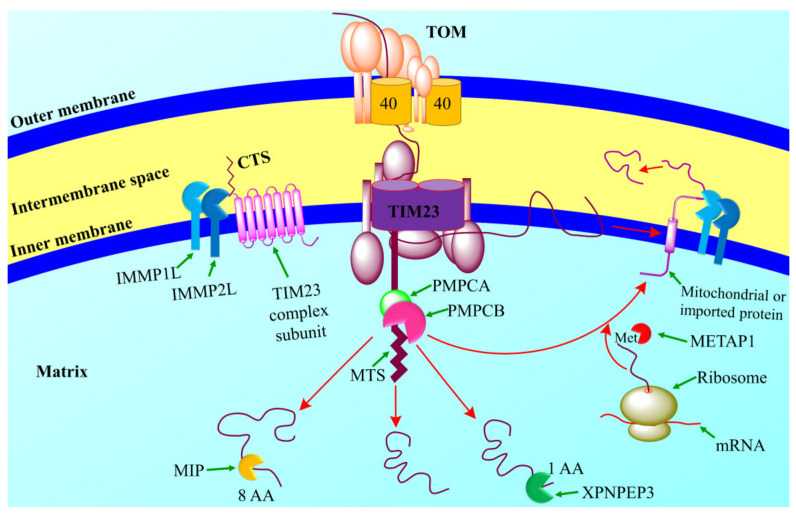
The mechanism of action of mitoprotease peptidases. Mitoproteases are the diverse group of enzymes that transport the biologically active proteins from cytosol to the inner mitochondrion to carry out the correct signals necessary for cellular processes [182]. Mitochondrial processing peptidase (MPP) is the part of the mitochondrial matrix that comprises two subunits such as PMPCA and PMPCB (protease mitochondrial processing peptidase subunit a and b) [183]. Mitochondrial intermediate peptidase (MIP) promotes the cleavage of octapeptide and X-Pro aminopeptidase 3 (XPNPEP3) that removed the amino acids from the amino-terminal of the MPP. Some proteins also pass through further photolytic cleavage via IMMP1L and IMMP2L. IMMPs attack the carboxy-terminal sequence (CTS) to promote cellular assembly [184]. Met aminopeptidase 1D (METAP1D) attacks on amino terminals of the initial Met of some of the polypeptides in order to get functionally active proteins [180]. This figure is reproduced from Quiros et al. [181] after permission from Springer Nature (License No. 5197691283486).

Mitoproteases mainly participate in the proteins trafficking into mitochondria from cytosol with the help of chaperones and help in the activation and folding of proteins imported into mitochondria [180]. Proteases are synthesized in the cytosol and move the processed proteins into mitochondria, which helps in the correct entry of proteins into mitochondria by removing mitochondrial import signals (see Figure 4) [178,181].

### 8.2. Cysteine Proteases in Atherosclerosis

**Figure 5:**
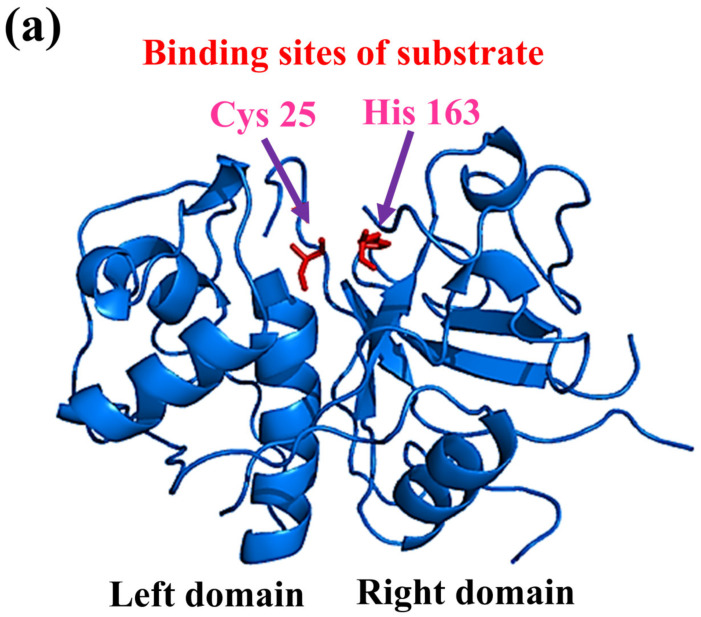

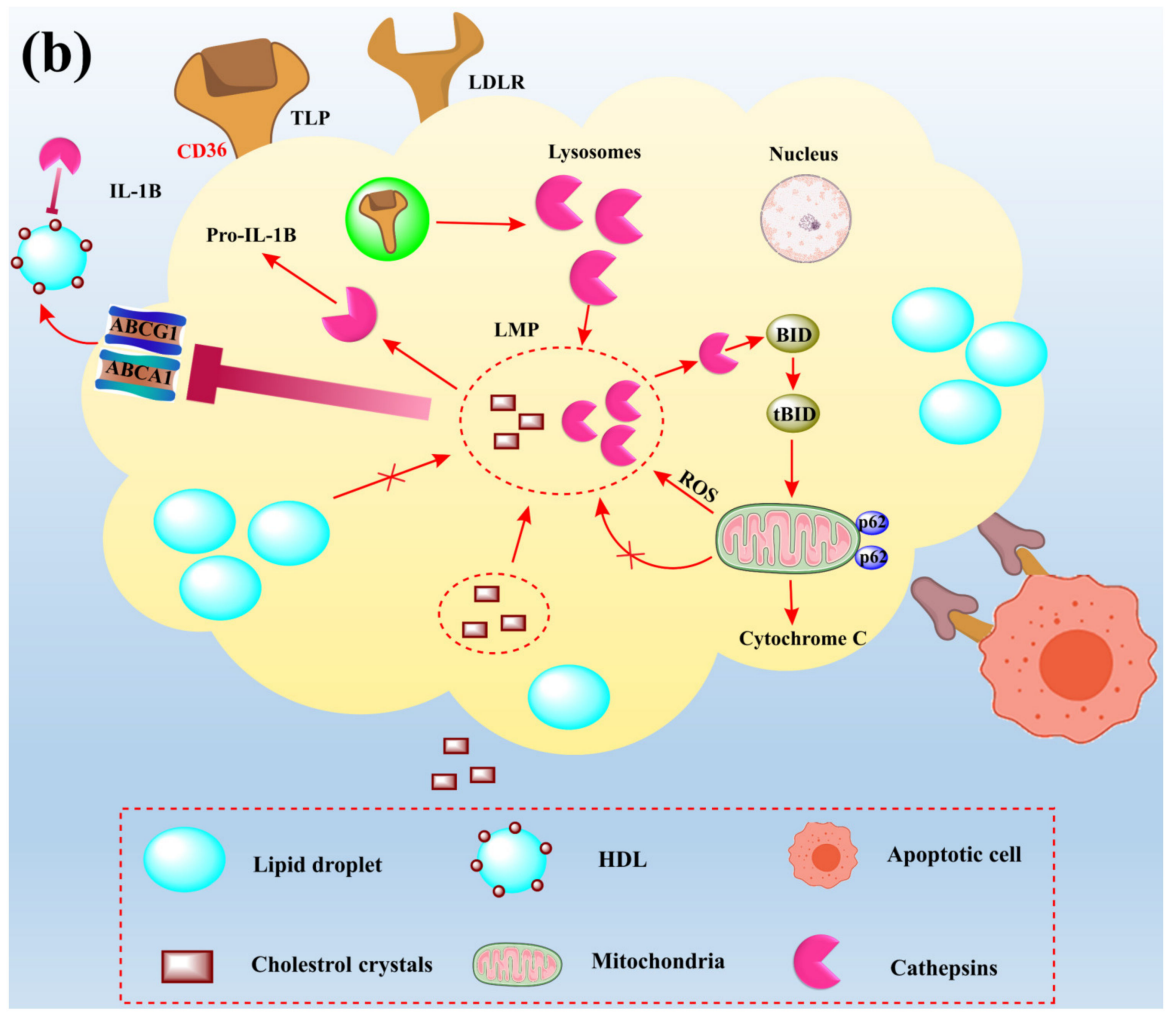
The representative structure of **(a**) fold of mature cathepsin L and (**b**) role of cathepsins in regulating inflammation in atherosclerosis. Cathepsins are involved in the regulation of inflammation in atherosclerosis by clearing the apoptotic cell [187]. Cathepsin function in atherosclerosis is catalyzed by suppressing the oily foam formation that leads to atherosclerosis in arteries due to excess cholesterol that increases the chances of reactive oxygen species and hence strong activity by clearance of the apoptotic cell. Cathepsins move into the cytosol by promoting apoptosis by degrading excess high-density lipoproteins (HDL) by processing the IL-1β [188,189]. Lysosomes bind to Low-density lipoprotein receptors (LDLR), causing them to degrade fatty acids and lipids through acid hydrolases. Impairment in cathepsin’s catalytic functioning leads to the deposition of excess cholesterol that causes stress and free radicals due to reactive oxygen species [185]. tBID: Truncated BID, LMP: Lysosomal membrane permeabilization, TLR: Toll-like receptor, ABCA1: ATP-binding cassette transporter, ABCG1: ATP-binding cassette sub-family G member 1, BID: BH3-interacting domain death. This figure is reproduced from Weiss-Sadan et al. [185] (Attribution 4.0 International, CC BY 4.0).

Analysis of human tissues has determined that overexpression of cathepsin activity can lead to various inflammatory conditions, including rheumatoid arthritis and atherosclerosis [185]. During atherosclerosis, cathepsins’ overexpression can be found in almost every cell that contains plaque tissues, mainly in macrophages and endothelial cells (see Figure 5) [186].

### 8.3. Role of Fungal Proteases in the Pathogenesis of Chronic Rhinosinusitis with Nasal Polyps

**Figure 6:**
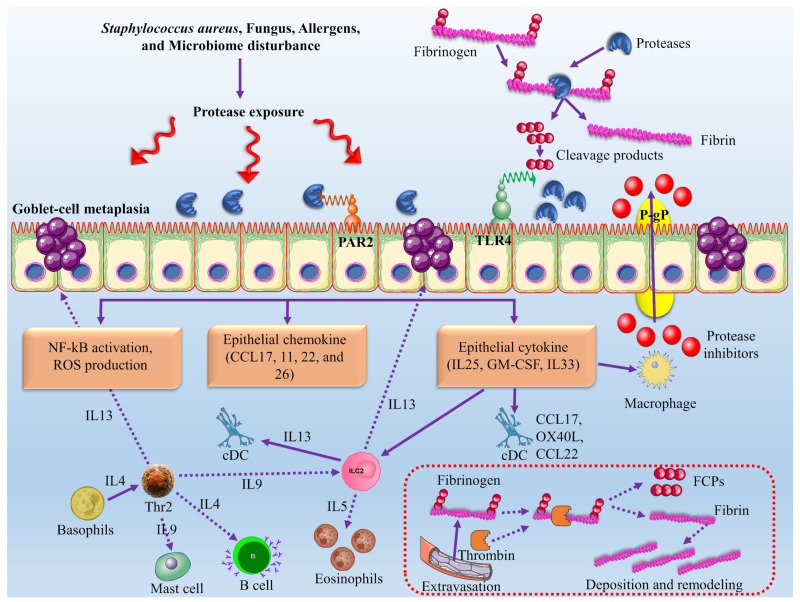
The mechanism of action of proteases to inhibit the allergen and other microbe attacks. Proteases are released in response to allergens due to a microbial attack that causes severe damage to epithelial cells by disrupting the junctional proteins [192]. Allergen proteases bind to protease-activated receptor 2 (PAR2) by cleaving the fibrinogen into the fibrin cleavage products (FCPs) that activate the toll-like receptor 4 (TLR4) and ILC2s. These ILC2s lead to activation of NF-kB and excess production of reactive oxygen species (ROS). It activates epithelial cells to release the pro-Th2 cell chemokines and cytokines that activated the instruct immature dendritic cells (iDCs). P-glycoproteins (P-gp) helps in removing the protease inhibitors in epithelial cells that cause the suppression of allergen proteases [190,193]. This figure is reproduced from Wu et al. [190] (Attribution 4.0 International, CC BY 4.0).

*Staphylococcus aureus*, fungi, cockroaches, and some airborne mites have serine proteases [190]. Interaction of proteases derived through allergens occurs with epithelial cells through three pathways: direct effects of protease on junctional proteins, epithelial activation through toll-like receptor 4, and reaction with protease-activated receptors present on the cell surface (see Figure 6) [191]. 

### 8.4. Fungal Protease as a New Therapeutic Strategy for Colorectal Cancer

Colorectal carcinoma is the most common cancer and has a high prevalence rate in developed countries due to issues related to diet. In the past decades, despite several drugs being used to treat colorectal cancer, the disease under the metastatic stages remains lethal. New enzymes therapy is urgently needed to treat the defective proteins during cell cycle stages. Proteases have been used for the suppression of tumour formation at the cellular level. A ubiquitin-proteasome system is a protease-based therapy for colorectal cancer [194]. 

The ubiquitin-proteasome system is the multiplex protease enzyme system essential for the survival of normal cells that causes the degradation of misfolded proteins. The ubiquitin-proteasome system also plays a critical role in controlling the turnover of the aberrant proteins or short-lived regulatory proteins involved in essential cellular processes, cell death, and cell signalling pathways, apoptosis, metastasis, and cell proliferation [195]. Protein substrates are mainly linked to the chain of the protein ubiquitin in the presence of ubiquitin-activating enzymes or E1, ubiquitin-conjugating enzymes or E2, and ubiquitin ligases or E3 to form the ubiquitin linkage. Polymerized ubiquitin chain provides the signal to transport the target proteins to the proteasome, proteolysis of the aberrant or target proteins broken down [196]. 

### 8.5. Protease as a New Therapeutic Strategy for Coeliac Disease

Celiac disease is one of the autoimmune disorders of the digestive system that affects the small intestine due to improper digestion of gluten-based toxic peptides such as gliadin. Recently, different protease-based therapies with a healthy diet plan and a gluten-free diet have effectively treated celiac disease [197]. Microbial peptidases also have been used to detoxify gluten by digesting them at the glutamine and proline sites. This approach cannot be practical due to the inactivation of most enzymes in the stomach wall through acidic pH [198].

Protease therapy is the best approach for protecting patients who have celiac disease from toxic or unwanted peptides. A recent study investigated the therapeutic use of the protease inhibitor elafin for the treatment of celiac disease [199]. Patients who have celiac disease expressed a lower amount of protease inhibitor elafin. Researchers performed experiments on mice models and found that delivery of elafin could reduce the inflammation in the small intestine. Elafin also protected the stomach wall by inhibiting the transformation of the gliadin peptides into the less toxic form [200].

### 8.6. Protease as a New Therapeutic Strategy for Neurological Disorders

Different disorders caused by prions are currently untreatable and universally fatal. Prions are the misfolded proteins that cause different fatal diseases in animals such as Gerstmann–Straussler–Scheinker syndrome and Creutzfeldt–Jakob disease [201]. Gerstmann–Straussler–Scheinker syndrome is a neurodegenerative disorder characterized by mutations in the PRNP gene due to prions that affect brain tissues. Creutzfeldt–Jakob disease is also a neurodegenerative disorder characterized by high prions in the nervous tissues that ultimately cause irreversible damage to the nerves. Prions disrupt brain functioning by changes in the neural tissues [201,202]. 

Enzyme-based therapy using the proteases is helpful for degrading the misfolded proteins that cause alterations in brain tissues. Recently, the *Bacillus* strain was successfully isolated and purified and was helpful in degrading the scrapie PrP^Sc^. *B. pumilus* KS12 produces the keratinase that plays a vital role in degrading the Sup35NM. Genetically engineered protease (MSK 103) is also effective against the PrP^Sc^. However, novel proteases should be discovered that can be used for targeting the prion diseases in animals [203,204]. 

## 9. Industrial Applications

Fungul protease has many applications in different sectors such as medicines and food industries, and it acts as a magic tool in biological research. It has diverse applications at industrial scales. Due to specificity in its mechanism of action, the protease is primarily involved in the formulations of medicines [205].

### 9.1. Food Industry

**Figure 7:**
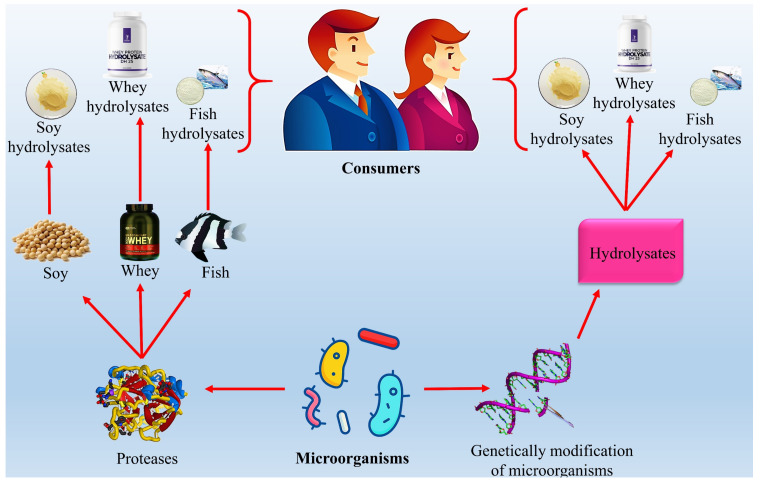
The application of fungal proteases in the food industry.

Proteases are used in food industries to manufacture wine, bread, cheese, and butter. Proteases in the dairy industry mainly act on the peptide bonds in cheese. Acid protease is used for the production of ethanol, which can be used as a source of nutrients for yeast. Acid protease acts on peptide bonds among amino acids, thus increasing the formation of ethanol from protease [90]. It resulted in the formation of casein, a protein found in milk. Some fungal strains such as Mucor michei are used to produce acidic proteases that can be used in cheese formation, thus replacing renin. Alkaline proteases are used in soya sauce, and enzymatic reactions that lead to the production of a high-quality protein called hydrolysates have different applications in nutrients and dairy-based products (see Figure 7) [15]. Fungal proteases that are used in the fruit juice and beverage industry mainly act on the peptide bonds among the proteins by degrading the complex compounds. It formed a turbidity complex from protein that ultimately improved gelatin hydrolysis, whey protein hydrolysis, gelatin hydrolysis, and meat mineralization [109]. Fungal-based proteases are utilized in food industries due to high specificity and catalytic activities as compared to the other fungal-based enzymes [176]. 

### 9.2. Waste Management and Bioremediation

Poultry industry waste and animal feathers could cause the deaths of livestock and other animals. Alkaline serine proteases are used to degrade keratin protein in the poultry industry [206]. Keratin is a structural protein with a long chain of amino acids linked through hydrogen and hydrophobic interaction [207]. Keratin released from the poultry industry releases waste and hazardous chemicals that can be controlled; alkaline proteases mainly act on the hydrogen bonds and hydrophobic linkages among the keratin [208]. It helps control the waste and hence plays a vital role in controlling environmental pollution. Different strains of fungi are used for degrading the keratin, namely *Pseudomonas* sp., *Aspergillus oryzae*, *Fusarium oxysporum*, *Trichophyton* sp., *Aspergillus terreus*, and *Scopulariopsis* sp., [209].

Fungal enzymes also play an essential role in bioremediation to control environmental pollution. Different strains of fungi such as *Hanerochaete chysosporium*, and *Pleurotus* sp. are used for bioremediation to maintain the ecological pollutant, thus helping in reducing the pollution [210]. Other applications of alkaline protease are found in degrading toxic proteins and chemicals released through the household. This alkaline protease acts on the toxic proteins and helps them to degrade into smaller fragments that can be released into the environment [211]. Thus, alkaline protease helps clean the environment by controlling the pollution from different sources of land and poultry through fungal-specific strains. 

### 9.3. Medical and Pharmaceutical Industry

In medicine, some fugal proteases are isolated from *Paecilomyces marquandii* and *Doratomyces microsporus* as source of potent keratinases that might be utilized in the elimination of keratin in acne or psoriasis, and degradation of keratinized skin, depilation, preparation of vaccine for dermatophytosis therapy, and in the increase of fungal drug delivery [212,213]. These keratinases can remove the scar and regenerate the epithelia, accelerate healing processes, and might act also in the medicine of trauma. A new semi-alkaline protease with high collagenolytic activity was produced by *Aspergillus niger LCF9*. The enzyme hydrolyzed various collagen types and liberated low molecular weight peptides of potential therapeutic use [212]. 

Thus, the demand for fungal proteases increases in the pharmaceutical and medical fields. Fungal aspartic proteases from *A. niger* are used for the preparation of digestive powders [22]. L-glutaminase from *Aspergillus fumigatus* and *Penicillium allii* is used for the treatment of leukaemia by inhibiting the growth of cancerous cells [214]. Alkaline protease is also used as a digestible in the pharmaceutical industry to treat cystic fibrosis [215,216]. These proteases control the abnormal concentrations of bile salts and pancreatic selections [109]. Alkaline protease is used to treat the cancerous cells as these enzymes particularly target the fibrin and promote their degradation, thus they are used as an anticancer agent in thrombolytic therapy [90]. Matrix metalloproteinase catalyzes the breakdown of the peptide bond in the clot formation molecule by adding a water molecule. These proteins showed the expression against tumor cells [217].

### 9.4. Proteases in Silver Recovery

X-ray and photographic films are generally composed of 2% silver embedded in the gelatin layer. This silver can be recovered through different approaches. Conventionally used methods retrieved the silver recovery through the burning of the X-ray films that cause environmental issues. It also increased the risk of respiratory infections in nearby areas due to the explosion of carbon monoxide. Therefore, conventionally used methods for silver recovery are not effective, and an urgently advanced approach is needed to reduce the environmental and safety issues [218]. Alkaline proteases have been effectively used for silver recovery with no environmental hazards. Proteases from the *Bacillus* species can catalyze the hydrolysis of the gelatin layer with maximum retrieval of the silver by ensuring no damage from polyester base film that can be recycled [219]. 

### 9.5. Proteases in Silk-Degumming

Silk fibers are covered by sericin, the fibrous protein that surrounds them in the form of rough texture. Different conventional methods have been used for the removal of sericin in order to organize the structure of silk fibers [220]. These methods are expensive due to the high-cost machinery required to remove the sericin. Therefore, conventionally used methods for silk-degumming are not reliable. Proteases have been used for degumming silk in order to remove the sericin with the maintenance of fiber structure. Alkaline protease obtained from the *Bacillus* sp., RGR-14 is used for degumming silk prior to the conventional methods [221].

## 10. Novel Protease Inhibitors

Different inhibitors have been isolated and purified in searching for novel proteasse inhibitors—the novel protease inhibitor, PPF-BBI, that is separated from the skin of *Pelophylax fukienensis*. PPF-BBI as a novel inhibitor showed antimicrobial activity against *E. coli* and *C. albicans* [222]. Another study investigated the role of the novel serine protease inhibitor GP205 in the NS3/4A protein. The pharmacological analysis of the GP205 inhibitor showed the biological activities in targeting the HCV virus, and ultimately, this novel inhibitor could be a possible treatment for the Hepatitis C virus [223]. Different compounds quercetin 3-β-d-glucoside, helichrysetin, and herbacetin have been screened by flavonoid library. The biochemical analysis of these compounds showed biological activities in suppressing the MERS-COV 3Cl protease. These chemical compounds could be used as a possible treatment in targeting coronaviruses [224].

There are several protease inhibitors that showed broad spectrum inhibitory specificity and antifungal activity by tight binding [225] (see Table 5). These inhibitors are survivin (cysteine inhibitor) [226,227], diosgenin (metalloprotease inhibitor) [228,229], serpin (serine inhibitor) [230], saccharopepsin (aspartic acid inhibitor) (IA3) [231], streptomyces (metallopeptidase inhibitor; SMI) [232], RFLP-1 (Rhamnus frangula inhibitor) proteases [233]. These protease inhibitors have gained special importance in biomedicine. 

### 10.1. Protease Inhibitors in Clinical Trials

#### 10.1.1. HIV

Protease inhibitors have been used as therapeutic agents to target the human immunodeficiency virus that usually attacks immune cells. FDA has approved many protease inhibitors for HIV. These inhibitors included ritonavir, amprenavir, and indinavir. These inhibitors bind to HIV and inhibit viral replication [234,235]. Some protease inhibitors are currently under clinical trials; RO033-4649 (Roche) entered the phase I stage and TMC-114 (Tibotec) is under clinical phase III. These therapeutic agents have reduced the mortality rate and are helpful for treatment [236,237,238]. 

#### 10.1.2. HCV

Hepatitis C virus has become the most common cause of liver cirrhosis. Many protease inhibitors for HCV are currently in the clinical trials stage. These inhibitors include SCH 6 (Shering), phase I, and VX-950 (Vertex), which has entered the clinical trial phase II [239].

#### 10.1.3. Cancer

Protease inhibitors have been designed for the treatment of cancer. They are currently used as therapeutic agents to control the different mutations. Many protease inhibitors are currently in clinical trials. These inhibitors are COL-3 (Collagenex), which has entered the phase II stage and AG3340 (Agouron), which is in phase III [240,241]. 

### 10.2. Mechanisms of Action for Protease Inhibitors

**Figure 8:**
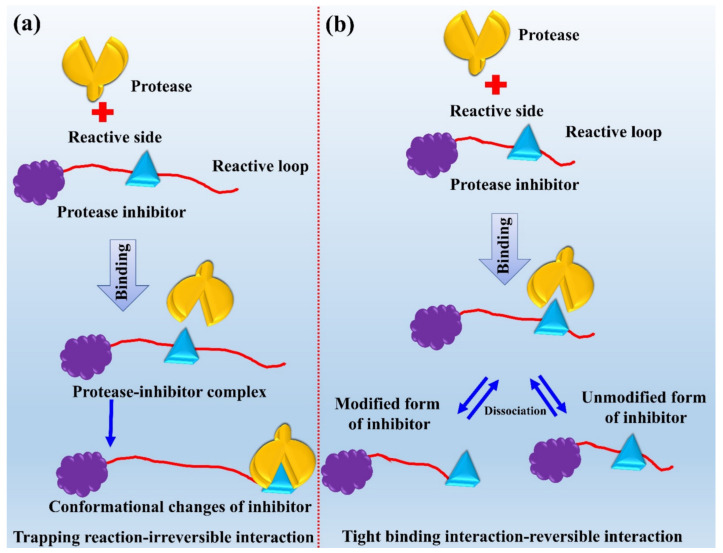
Mechanism of action of fungal protease inhibitors. (**a**) Trapping reaction-irreversible interaction and (**b**) Tight binding interaction-reversible interaction. This figure is reproduced from Rudzinska et al. [216] (Attribution-NonCommercial 3.0 Unported, CC BY-NY 3.0).

Inhibitors are those molecules that inhibit the activity of the proteases by blocking them. It can be explained via two different mechanisms (see Figure 8). In the case of a reversible reaction called tight binding interaction between protease and inhibitor, the inhibitor binds to the protease’s active site. It results in forming a protease-inhibitor complex that can be broken down into the modified and unmodified form of the inhibitor. In the case of irreversible reaction that is also called trapping interaction between protease and inhibitor, the inhibitor binds to the protease’s active site. This results in the cleavage of the peptide bond of the inhibitor and causes the conformational change of the inhibitor [242,243]. 

### 10.3. Discovery of Protease Inhibitors against COVID-19

Coronavirus Main protease (Mpro) inhibitors receive great attention in targeting COVID-19 because of their role in processing the replicase during the post-translational processes. Their active site comprises two catalytic regions, C145 and H41 [244]. PLpro has been involved in bioprocessing the viral polypeptides into functional proteins. Their active site is composed of three catalytic regions including C111, H272, D286 [244]. These two proteases play a vital role in transcription by processing the two polypeptide proteins, pp1a and pp1ab, of the *Coronaviridae* genome. Therefore, these proteases can be used for antiviral drugs and discovery. These inhibitors possess poor absorption, excretion problems, and toxicological effects. Hence, the need to discover protease inhibitors remains a challenge [245]. 

In the current scenario, approaches for fragment-based drug design can help design protease inhibitor candidates that follow the screening and identification of those compounds with low molecular weight, advanced computational methods for the identification and sequencing of good fragments from the peptidomimetic compounds, and optimization of the [246] potential coronavirus protease inhibitor candidate against the COVID-19 [247,248].

The potential roles of Mpro and PLpro inhibitors for the treatment of SARS-CoV-2 are under clinical trials. For designing possible protease inhibitors for COVID-19, only those inhibitors that can inhibit the early viral replication by binding with the original substrate can reach the local target sites and provide high bioavailability. These inhibitors must be tested for antiviral activity through cellular and molecular pathways [249]. 

### 10.4. NP-Delivery Systems for Discovery of Protease Inhibitors

Most of the protease inhibitors have been tested for clinical trials for the treatment of cancers. These inhibitors included the prinomastat, tanomasta, and neovastat. These inhibitors have developed poor efficacy for clinical outcomes. Conventional chemotherapies for cancer treatment are not reliable due to poor diagnosis of cancerous cells at an early stage of cancer [216,250]. However, present challenges include designing protease inhibitors with a formulation of different types of nanoparticles for the treatment of cancers. Tumor cells also show chemoresistance to the conventionally developed chemotherapeutic agents. Protease inhibitors are promising candidates combined with NP-based delivery systems that can overcome chemoresistance [216].

Through advances in nanotechnology, the protease inhibitors carfilzomib and bortezomib have been designed with NP-delivery systems such as gold, PEGlycated, silica, and liposomes. These combinations of protease inhibitors demonstrate a high efficacy rate, increased circulation to the target cells, biocompatibility, and decreased systemic toxicity for breast, colon, and lung cancer [251,252]. This nanotechnology-based approach could help reduce the side effects of drugs, protect the normal tissues, and improve patients’ quality. The detailed information about protease inhibitors with mechanism is presented in Table 6.

## 11. Current Challenges and Future Perspective

Fungal proteases can be used as a target for therapeutic interventions because they are involved in many pathologic processes. Fungal proteases are challenging to target due to small information about substrate specificity and natural inhibitors [20]. The development of advanced techniques such as activity-based probes allows monitoring protease activity in living cells and helps understanding of protease function [253]. Fungi produce a wide variety of proteolytic enzymes used in biotechnology, food, detergents, and soil bioremediation.

The complete multiple genome sequencing has enabled us to identify the different classes of proteases, but there are still many gaps in understanding the role of many proteases [254]. Some significant challenges are addressed by the protease group in the development and usage of technologies at GNF in understanding protease biology to develop protease-based targeted therapeutics [255]. The GNF protease platform enables the use of comprehensive technologies that help identify native and optimal substrates [256]. Due to industrial development, environmental pollution has become a major concern, and chemically thermostable protease has taken over the conventional protease [257]. Toxic chemicals, including sodium sulphide, salt, lime, and solvents, are released from these industries and can pollute the environment [210,258]. Chemical industries are the first target to eliminate environmental pollution, and pre-tanning procedures produce a higher amount of pollution than post-tanning procedures [259].

The industrial and therapeutic use of proteases has grown in the past two decades. The expansion in protease markets has occurred due to unique protein engineering techniques [260]. For clinical applications, the success of apoptotic caspase activation with small, engineered protease can control the activity of the human protease. Due to proteolytic activities, tumor-imaging and drug-targeting a specific site can be enhanced in disease tissues with the help of proteases. Due to advancements in screening techniques, crystallography, and synthetic biology, we can hope that in the future, proteases will gain dramatic success [257,261].

## 12. Conclusions

Enzymes from fungi are used in a wide range of industries, including the pharmaceutical and agricultural sectors. Using enzymes in many industrial applications is a more cost-effective and ecologically friendly alternative. It is common practice in the biotechnology industry to employ fungi-derived proteolytic enzymes in the food and leather industries, as well as in ecological bioremediation processes, and to manufacture medicinal peptides. All living creatures rely on fungi and their proteases for a wide range of physiological, metabolic, and regulatory functions. Since fungal proteases are implicated in a wide range of diseases, they might be useful therapeutic targets. For fungal proteases, the lack of knowledge on substrate specificity and endogenous and natural inhibitors makes it challenging to build selective inhibitors. Transcriptional control of fungal extracellular protease expression may be a good candidate for genetic modification. CRISPR/Cas9 technology is anticipated to play a bigger role in genetic modification studies for filamentous fungus in the future as the technology continues to advance. Tools for functional genomics in filamentous fungal species will make it easier to implement fast advancements in CRISPR gene-editing technology. 

## Figures and Tables

**Table 1:** The characteristics of different fungal enzymes.

Enzyme Class	Type of Strain	Production Method	Mol. Weight (kDa)	Opt. pH	Opt. Temperature (℃)	Substrate	Inhibitors	Enzyme Activity (%)	Reference
Fungal amylase	*Thermomyces lanuginosus*	SSF	33	5	48	Wheat straw, guayule bagasse	SDS	88	[37,38]
	*Aspergillus fumigatus*	SmF	28	4.5	30	Pomegranate peel, wheat bran	Ebselen	90	[39,40]
	*Cryptococcus flavus*	SSF	70	5	45	Starch, amylose	Mercury	80–84	[41]
	*Aspergillus niger*	SSF	115	5	75	Cowpeas, chickpeas		88	[42,43]
	*Mucor* sp.	SSF	38	4	30	Kidney beans, lupine	EDTA	84–86	[44,45]
	*Aspergillus oryzae*	SSF	102	8	30	Groundnut oil, sesame oil	Copper	85	[46,47]
	*Aspergillus kawachii*	SSF	110	3	32	Pearl millet	Mercury	88–90	[48,49]
	*Penicillium fterreus*	SSF	25	6	28	Cowpeas, chickpeas	Lead	78	[43,50]
Fungal cellulase	*Trichoderma viride*	SSF	55	7	52	CMC	Mercury	86–88	[51,52]
	*Peniophora* sp.	SmF	30	4	58	SKT	EDTA	80	[53]
	*Aspergillusniger IMMIS1*	SSF	70	3.5	32	RW, bread	Mercury	90	[54,55]
	*T. harzianum*	SSF	40	5	70	Sugarcane bagasse	EDTA	85	[13,56]
	*Aspergillus niger VTCC-F021*	SSF	29	4	52	Sugar cane, CMC	Zinc	95	[57,58]
	*Aspergillus terreus*	SmF	52	3.5	13	Cowpeas, chickpeas	Mercury	78	[59]
Fungal lipase	*Aspergiillus niger*	SSF	30	3	40–48	Coir waste, RH	Zinc	88	[42,60]
	*A. terreus*	SmF	35	3.5	48	MOC	α-Glucosidase	85	[59,61]
	*A. versicolor*	SmF	93	7	60	EMO	Lipstatin	90	[62,63]
	*A. tamarii*	SSF	5	4.5	55	GOC, agrowastes	Ebelactone	92	[64,65]
	*A. japonicus*	SmF	9	4.6	25	SFO, casein	Caulerpenyne	88	[66,67]
	*Mucor* sp.	SmF	7	4.2	28	Kidney beans, lupine	Percyquinin	66	[45,68]
Fungal protease	*Scopulariopsis* sp.	SSF	38	8	56	Glucose, peptone	NBS	88	[69,70]
	*Aspergillus niger*	SSF	47	7	45	Cowpeas, chickpeas	EDTA	88	[42,71]
	*Aspergillus fumigatus*	SSF	40	8	31	PL, casein	DTT	90	[72,73]
	*Rhizopus oryza*	SmF	55	5	32	WBW	DTT	75	[74,75]
	*Mucpr* Sp.	SSF	35	7	28	Kidney beans, lupine	EDTA	88	[45,76]
	*G. putredinis*	SmF	48	7–8	29	Soya bean meal	IAA	85	[13]
	*T. harzianum*	SSF	45	7	19	Glutamine	PMSF	80–82	[13]

Note: SDS = Sodium Dodecyl Sulfate, EDTA = Ethylenediaminetetraacetic acid, DTT = Dithiothreitol, NBS = *N*-bromosuccinimide, PMSF = Phenylmethylsulphonylfluoride, IAA = Indole Acetic Acid, SSF = Solid-state fermentation, SmF = submerged fermentation, RH = Rice Husk, MOC = Mustard Oil Cake, GOC = Gingili Oil Cake, EMO = Edible Oil Mill, SFO = Sunflower Oil, PL = Pig Hung, CMC = Carboxy Methyl Cellulose, SCT = Silk Cotton Tree, RW = Rose Wood, and WBW = White Bread Waste.

**Table 2:** The coproduction of fungal enzymes from different strains of fungus based on temperature.

Fungal Strain	Nature	Genus	Opt. Temperature (°C)	Fungal Amylase	Fungal Cellulase	Fungal Lipase	Fungal Protease	Application	Reference
*Thermomyces lanuginosus*	*Thermophilic fungus*	*Thermomyces*	40–50	√	✕	√	√	Wastewater and pharmaceuticals waste treatment	[81,82]
*Sporotrichum thermophile*	*Thermophilic fungus*	*Sporotrichum*	45–50	✕	√	✕	√	Biomass degradation	[86,87]
*Myceliophthora thermophila*	*Thermophilic fungus*	*Myceliophthora*	45–50	✕	√	✕	√	Textile industries and bioremediation	[84]
*Thermomyces ibadanensis*	*Thermophilic fungus*	*Thermomyces*	46–55	✕	✕	√	√	Wastewater treatment	[85]
*Neurospora crassa*	*Mesophilic fungus*	*Neurospora*	20–30	✕	√	✕	√	As a model organism in an analysis of genetic recombination	[83]
*Aspergillus niger*	*Mesophilic fungus*	*Aspergillus*	20–30	√	✕	✕	√	Food industries	[88]
*Aspergillus flavus*	*Mesophilic fungus*	*Aspergillus*	25–30	√	√	✕	√	Textile, detergent, and paper industries	[88]
*Candida mogii*	*Psychrophilic fungus*	*Candida*	5–10	√	✕	√	√	Food industries	[89]

Note: The tick (√) represents ‘produce’, and cross (✕) represents ‘not produce’.

**Table 3:** The representation of novel fungal protease enzymes isolated from different sources.

Enzyme Isolated	Enzyme Class	Active Site Residue (s)	Isolated Source	Reference
Clostripain, Streptopain	Cysteine proteases	Cysteine and histidine residues	*C. histolyticum*, *S. griseus*	[98,99]
Pepsins, proteases, rennet like proteases	Aspartic endoproteases	Two aspartate residues	*A. niger*, *M. miehei*	[100,101]
Chymotrypsins, subtilisins	Serine proteases	Serine residues	*B. sphaericus*	[102,103]
Collagenases, elastase	Metalloendoproteases	Metal ions	*C*. *histolyticum*, *P. aeruginosa*	[104,105]
Eqolisin protease	Glutamic proteases	Glutamate residues	*S. lignicola*, *A. niger*	[106,107]
Pepsins (A1), retropepsin (A2)	Acidic proteases	-	*A. niger*, *A. saitoi*.	[108,109]
Subtilisin, carlsberg	Alkaline proteases	-	*A. salinivibrio*, *C. aureus*	[109]
Neutrase, thermolysin	Neutral proteases	-	*Bacillus* sp.	[109]

**Table 4:** The applications and efficiencies of different fungal enzymes isolated from fungal strains recently modified through CRISPR technology.

Fungal Strain	Isolated Proteases	CRISPR System	Selective Marker	Promoter for sgRNA	Promoter for Cas9	Delivery Method	Editing Method	Application	Efficiency (%)	Reference
*A. oryzae*	Aspartic acid protease	Cas-9	*pyrG*	U6	*amyB*	PMT	NHEJ	Genetic engineering, food industries	10–30	[158,159]
*M. thermophila*	Alkaline protease	Cas-9	*bar*	U6	*tef1*	AMT	HDR	pharmaceuticals biomass/waste treatment	20–95	[86,156]
*T. lanuginosus*	Serine alkaline protease	Cas-9	*hph*	U6	*act1*	AMT	NHEJ	Wastewater and pharmaceuticals waste treatment	44–90	[82,160]
*C. militaris*	Serine alkaline protease	Cas-9	5-FOA/blpR	U6	*tef1*	AMT	NHEJ	Pharmacecurials	82–89	[161,162]
*F. graminearum*	Acid proteases	Cas-9	*Fludioxonil*	U6	*gpdA*	AMT	HDR	Food indsuries, pests conrol	2–12	[163,164]
*U. maydis*	Metalloproteases	Cas-9	*Ip*	U6	*otef*	PMT	NHEJ	Biofuels, pharmacuetcals	50–90	[165,166]
*N. crassa*	Serine proteases	Cas-9	*bar*	SNR52	*TrpC*	AMT	HDR	Genetic recombination	60–80	[83,167]
*G. lucidum*	Metalloproteases	Cas-9	*ura3*	T7	*gpdA*	PMT	NHEJ	Medicine	28–33	[168,169]

Note: NHEJ = Non-homologous end joining, HDR = High-fidelity homology-directed repair, Cas = CRISPR-associated genes, gRNA = Guide RNA, sgRNA = Single guide RNA, AMT = Agrobacterium mediated transformation, and PMT = Protoplast transformation.

**Table 5:** Fungal protease inhibitors for biomedical applications.

Type of Fungal Protease Inhibitors	Proteases Inhibited	Family	Activity	Inhibitory Mechanism	Application	Reference
Survivin (Cysteine inhibitor)	Caspases- specific cysteine proteases	C14	Antifungal	Tight binding	Role as a mitotic regulator of cell division and as an inhibitor of caspase activation in the process of apoptosis.	[226,227]
Diosgenin (Metalloprotease inhibitor)	Metalloproteases	M15	Antifungal	Tight binding	These inhibit the secreted metallopeptidase relevant in brain invasion by cryptococcal cells, causing meningoencephalitis.	[228,229]
Serpin (Serine inhibitor)	Serine Proteases	C1 and C14	Antifungal	Trapping traps the serine protease in a covalent complex	Fungal serpins as a therapeutic benefit toward several inflammation-related complications.	[230]
Saccharo (Pepsin, aspartic acid inhibitor) (IA3)	Aspartic acid proteases	C1	Antifungal	Tight binding	Role as an inhibitor in the process of apoptosis and cancer.	[231]
Streptomyces (Metallopeptidase inhibitor)	Metalloproteases	C14	Antifungal	Tight binding	Role as an inhibitor in tumor invasion and metastasis (the most validated target for cancer).	[228,232]
RflP-1 (Rhamnus frangula inhibitor proteases)	Serine protease	C14	Antifungal	Trapping	Role in free radical scavenging activities.	[233]

**Table 6:** Representation of protease inhibitors under clinical trials with the mechanism of action and therapeutic uses.

Name of Inhibitor	Targeted Enzyme	Target Disease	Clinical Trials Stage	Reference
RO033-4649	Therapeutic agents have reduced the rate of mortality and are helpful for treatment	AIDS	Under the clinical phase I	[238]
VX-950	Become the most common cause of liver cirrhosis	HCV	Under the clinical trial phase II	[239]
COL-3	Therapeutic agents to control the different mutations in colon cancer	Colon cancer	Entered the phase II stage	[240]
AG3340	Therapeutic agents to control the different mutations in lung cancer	Lung cancer	Entered the phase II stage	[241]
TMC-114	Therapeutic agents have reduced the rate of mortality and are helpful for the treatment	AIDS	Under clinical phase III	[237]
Indinavir	These inhibitors bind to the HIV and inhibit the viral replication	HIV	FDA has approved this inhibitor	[234,235]
Ritonavir	These inhibitors bind to the HIV and inhibit the viral replication	HIV	FDA has approved this inhibitor	[234,235]
GP205	GP205 inhibitor showed the biological activities in targeting the HCV virus, and ultimately, this novel inhibitor could be for possible treatment for Hepatitis C virus	HCV	GP205 inhibitor showed the biological activities in targeting the HCV virus, and ultimately, this novel inhibitor could be for possible treatment for Hepatitis C virus	[223]
Quercetin 3-β-d-glucoside and helichrysetin	The biochemical analysis of these compounds showed biological activities in the suppression of the MERS-COV 3Cl protease	Coronaviruses	These chemical compounds could be used as a possible treatment in targeting the coronaviruses	[224]
Mpro inhibitors	These inhibitors play a role in processing the replicase during the post-translational process’s viruses of the *Coronaviridae* family	Coronaviruses	These proteases can be used for antiviral drug and discovery	[244,245,246]
PLpro inhibitors	These inhibitors play a vital role in transcription by the processing of the two polyproteins, pp1a and pp1ab	Coronaviruses	These proteases also can be used for the discovery of novel protease inhibitors	[244,245,246]
NP-delivery systems-based carfilzomib and bortezomib	This nanotechnology-based approach could help reduce the side effects of drugs	These protease inhibitors can be designed with combinations with gold, PEGlycated, silica, liposomes and demonstrate the high efficacy rate	Future discovery of novel inhibitors based on modified NP-systems protecting the normal tissues and improving the quality of patients	[252]

## Data Availability

Not applicable.

## References

[1] Monod M., Capoccia S., Léchenne B., Zaugg C., Holdom M., Jousson O. (2002). Secreted proteases from pathogenic fungi. Int. J. Med. Microbiol..

[2] Singh R., Mittal A., Kumar M., Mehta P.K. (2016). Microbial proteases in commercial applications. J. Pharm. Chem. Biol. Sci..

[3] Rao M.B., Tanksale A.M., Ghatge M.S., Deshpande V.V. (1998). Molecular and biotechnological aspects of microbial proteases. Microbiol. Mol. Biol. Rev..

[4] Singh N., Gaur S. (2021). GRAS Fungi: A New Horizon in Safer Food Product. Fungi in Sustainable Food Production.

[5] Novelli P.K., Barros M.M., Fleuri L.F. (2016). Novel inexpensive fungi proteases: Production by solid state fermentation and characterization. Food Chem..

[6] Souza P.M.d., Bittencourt M.L.d.A., Caprara C.C., Freitas M.d., Almeida R.P.C.d., Silveira D., Fonseca Y.M., Ferreira E.X., Pessoa A., Magalhães P.O. (2015). A biotechnology perspective of fungal proteases. Braz. J. Microbiol..

[7] Kumar A., Gautam A., Dutt D. (2015). Screening of fungal resources for the production of cellulases and xylanases. Biotechnol. J. Int..

[8] Lange L. (2017). Fungal enzymes and yeasts for conversion of plant biomass to bioenergy and high-value products. Microbiol. Spectr..

[9] Damare S., Raghukumar C., Muraleedharan U.D., Raghukumar S. (2006). Deep-sea fungi as a source of alkaline and cold-tolerant proteases. Enzym. Microb. Technol..

[10] Kumar A. (2020). Aspergillus nidulans: A Potential Resource of the Production of the Native and Heterologous Enzymes for Industrial Applications. Int. J. Microbiol..

[11] Gnanadoss J.J., Devi S.K. (2021). Optimization of nutritional and culture conditions for improved protease production by Aspergillus nidulans and Aspergillus flavus. J. Microbiol. Biotechnol. Food Sci..

[12] Hoffmeister D., Keller N.P. (2007). Natural products of filamentous fungi: Enzymes, genes, and their regulation. Nat. Prod. Rep..

[13] Savitha S., Sadhasivam S., Swaminathan K., Lin F.H. (2011). Fungal protease: Production, purification and compatibility with laundry detergents and their wash performance. J. Taiwan Inst. Chem. Eng..

[14] Liu E., Li M., Abdella A., Wilkins M.R. (2020). Development of a cost-effective medium for submerged production of fungal aryl alcohol oxidase using a genetically modified Aspergillus nidulans strain. Bioresour. Technol..

[15] Sharma M., Gat Y., Arya S., Kumar V., Panghal A., Kumar A. (2019). A review on microbial alkaline protease: An essential tool for various industrial approaches. Ind. Biotechnol..

[16] Sabotič J., Kos J. (2012). Microbial and fungal protease inhibitors—current and potential applications. Appl. Microbiol. Biotechnol..

[17] Agbowuro A.A., Huston W.M., Gamble A.B., Tyndall J.D. (2018). Proteases and protease inhibitors in infectious diseases. Med. Res. Rev..

[18] Bezerra V.H.S., Cardoso S.L., Fonseca-Bazzo Y., Silveira D., Magalhães P.O., Souza P.M. (2021). Protease Produced by Endophytic Fungi: A Systematic Review. Molecules.

[19] Gupta V.K., Kubicek C.P., Berrin J.-G., Wilson D.W., Couturier M., Berlin A., Edivaldo Filho X., Ezeji T. (2016). Fungal enzymes for bio-products from sustainable and waste biomass. Trends Biochem. Sci..

[20] Yike I. (2011). Fungal proteases and their pathophysiological effects. Mycopathologia.

[21] Manganyi M.C., Ateba C.N. (2020). Untapped potentials of endophytic fungi: A review of novel bioactive compounds with biological applications. Microorganisms.

[22] Srilakshmi J., Madhavi J., Lavanya S., Ammani K. (2015). Commercial potential of fungal protease: Past, present and future prospects. J. Pharm. Chem. Biol. Sci..

[23] Adrio J.L., Demain A.L. (2003). Fungal biotechnology. Int. Microbiol..

[24] Guillemette T., van Peij N.N., Goosen T., Lanthaler K., Robson G.D., van den Hondel C.A., Stam H., Archer D.B. (2007). Genomic analysis of the secretion stress response in the enzyme-producing cell factory Aspergillus niger. BMC Genom..

[25] Nevalainen K.H., Te’o V.S., Bergquist P.L. (2005). Heterologous protein expression in filamentous fungi. Trends Biotechnol..

[26] Paloheimo M., Haarmann T., Mäkinen S., Vehmaanperä J. (2016). Production of industrial enzymes in Trichoderma reesei. Gene Expression Systems in Fungi: Advancements and Applications.

[27] Benito M.J., Connerton I.F., Córdoba J.J. (2006). Genetic characterization and expression of the novel fungal protease, EPg222 active in dry-cured meat products. Appl. Microbiol. Biotechnol..

[28] Li C., Xu D., Zhao M., Sun L., Wang Y. (2014). Production optimization, purification, and characterization of a novel acid protease from a fusant by Aspergillus oryzae and Aspergillus niger. Eur. Food Res. Technol..

[29] Salazar-Cerezo S., Kun R.S., de Vries R.P., Garrigues S. (2020). CRISPR/Cas9 technology enables the development of the filamentous ascomycete fungus Penicillium subrubescens as a new industrial enzyme producer. Enzym. Microb. Technol..

[30] Satish L., Shamili S., Muthubharathi B.C., Ceasar S.A., Kushmaro A., Singh V., Sitrit Y. (2020). CRISPR-Cas9 System for Fungi Genome Engineering Toward Industrial Applications. Genome Engineering via CRISPR-Cas9 System.

[31] Liu R., Chen L., Jiang Y., Zhou Z., Zou G. (2015). Efficient genome editing in filamentous fungus Trichoderma reesei using the CRISPR/Cas9 system. Cell Discov..

[32] Farooq M.A., Ali S., Hassan A., Tahir H.M., Mumtaz S., Mumtaz S. (2021). Biosynthesis and industrial applications of α-amylase: A review. Arch. Microbiol..

[33] Singh A., Bajar S., Devi A., Pant D. (2021). An overview on the recent developments in fungal cellulase production and their industrial applications. Bioresour. Technol. Rep..

[34] Mehta A., Guleria S., Sharma R., Gupta R. (2021). The Lipases and Their Applications with Emphasis on Food Industry. Microbial Biotechnology in Food and Health.

[35] Anitha T., Palanivelu P. (2013). Purification and characterization of an extracellular keratinolytic protease from a new isolate of Aspergillus parasiticus. Protein Expr. Purif..

[36] Zdarta J., Jędrzak A., Klapiszewski Ł., Jesionowski T. (2017). Immobilization of cellulase on a functional inorganic–organic hybrid support: Stability and kinetic study. Catalysts.

[37] Khan F.I., Bisetty K., Singh S., Permaul K., Hassan M.I. (2015). Chitinase from Thermomyces lanuginosus SSBP and its biotechnological applications. Extremophiles.

[38] Sikandar S., Ujor V.C., Ezeji T.C., Rossington J.L., Michel F.C., McMahan C.M., Ali N., Cornish K. (2017). Thermomyces lanuginosus STm: A source of thermostable hydrolytic enzymes for novel application in extraction of high-quality natural rubber from Taraxacum kok-saghyz (Rubber dandelion). Ind. Crops Prod..

[39] Dagenais T.R., Keller N.P. (2009). Pathogenesis of Aspergillus fumigatus in invasive aspergillosis. Clin. Microbiol. Rev..

[40] Singh S., Singh S., Bali V., Sharma L., Mangla J. (2014). Production of fungal amylases using cheap, readily available agriresidues, for potential application in textile industry. BioMed Res. Int..

[41] Wanderley K.J., Torres F.A., Moraes L.M., Ulhoa C.J. (2004). Biochemical characterization of α-amylase from the yeast Cryptococcus flavus. FEMS Microbiol. Lett..

[42] Stojanović J., Jakovljević V., Matović I., Gajović O., Mijušković Z., Nedeljković T. (2011). Influence of detergent on metabolic activity of fungi Aspergillus niger. Nat. Sci..

[43] Saleem A., Ebrahim M.K. (2014). Production of amylase by fungi isolated from legume seeds collected in Almadinah Almunawwarah, Saudi Arabia. J. Taibah Univ. Sci..

[44] Souza P.M.d., Magalhães P.d.O. (2010). Application of microbial α-amylase in industry-A review. Braz. J. Microbiol..

[45] Alves M.H., Campos-Takaki G.M., Porto A.L.F., Milanez A.I. (2002). Screening of Mucor spp. for the production of amylase, lipase, polygalacturonase and protease. Braz. J. Microbiol..

[46] Rahardjo Y.S., Weber F.J., Le Comte E.P., Tramper J., Rinzema A. (2002). Contribution of aerial hyphae of Aspergillus oryzae to respiration in a model solid-state fermentation system. Biotechnol. Bioeng..

[47] Balakrishnan M., Jeevarathinam G., Kumar S.K.S., Muniraj I., Uthandi S. (2021). Optimization and scale-up of α-amylase production by Aspergillus oryzae using solid-state fermentation of edible oil cakes. BMC Biotechnol..

[48] Saranraj P., Stella D. (2013). Fungal amylase—a review. Int. J. Microbiol. Res..

[49] Sethi B.K., Jana A., Nanda P.K., DasMohapatra P.K., Sahoo S.L., Patra J.K. (2016). Production of α-amylase by Aspergillus terreus NCFT 4269.10 using pearl millet and its structural characterization. Front. Plant Sci..

[50] Khajuria R., Singh S. (2020). Fungal Amylases for the Detergent Industry. Microbes in Agriculture and Environmental Development.

[51] Niyonzima F.N., More S. (2014). Purification and properties of detergent-compatible extracellular alkaline protease from Scopulariopsis spp.. Prep. Biochem. Biotechnol..

[52] Zahra T., Irfan M., Nadeem M., Ghazanfar M., Ahmad Q., Ali S., Siddique F., Yasmeen Z., Franco M. (2020). Cellulase Production by Trichoderma viride in Submerged Fermentation using Response Surface Methodology. Punjab Univ. J. Zool..

[53] Niyonzima F.N. (2020). Detergent-compatible fungal cellulases. Folia Microbiol..

[54] Imran M., Anwar Z., Zafar M., Ali A., Arif M. (2018). Production and characterization of commercial cellulase produced through Aspergillus niger IMMIS1 after screening fungal species. Pak. J. Bot..

[55] Imran M., Anwar Z., Irshad M., Javid A., Hussain A., Ali S. (2017). Optimization of cellulase production from a novel strain of Aspergillus tubingensis IMMIS2 through response surface methodology. Biocatal. Agric. Biotechnol..

[56] da Silva Delabona P., Lima D.J., Robl D., Rabelo S.C., Farinas C.S., da Cruz Pradella J.G. (2016). Enhanced cellulase production by Trichoderma harzianum by cultivation on glycerol followed by induction on cellulosic substrates. J. Ind. Microbiol. Biotechnol..

[57] Pham T.H., Quyen D.T., Nghiem N.M. (2012). Purification and properties of an endoglucanase from Aspergillus niger VTCC-F021. Turk. J. Biol..

[58] Pham T.H., Quyen D.T., Nghiem N.M. (2010). Optimization of endoglucanase production by Aspergillus niger VTCC-F021. Aust. J. Basic Appl. Sci..

[59] Niyonzima F.N., More S.S. (2015). Purification and characterization of detergent-compatible protease from Aspergillus terreus gr. 3 Biotech.

[60] Hernández M.S., Rodríguez M.R., Guerra N.P., Rosés R.P. (2006). Amylase production by Aspergillus niger in submerged cultivation on two wastes from food industries. J. Food Eng..

[61] Sethi B.K., Rout J.R., Das R., Nanda P.K., Sahoo S.L. (2013). Lipase production by Aspergillus terreus using mustard seed oil cake as a carbon source. Ann. Microbiol..

[62] Choudhary V. (2012). Production, isolation and characterization of alkaline protease from Aspergillus versicolor PF/F/107. J. Acad. Indus. Res..

[63] Gopinath S.C., Hilda A., Anbu P. (2005). Extracellular enzymatic activity profiles in fungi isolated from oil-rich environments. Mycoscience.

[64] da Silva O.S., de Almeida E.M., de Melo A.H.F., Porto T.S. (2018). Purification and characterization of a novel extracellular serine-protease with collagenolytic activity from Aspergillus tamarii URM4634. Int. J. Biol. Macromol..

[65] Dayanandan A., Rani S., Shanmugavel M., Gnanamani A., Rajakumar G.S. (2013). Enhanced production of Aspergillus tamarii lipase for recovery of fat from tannery fleshings. Braz. J. Microbiol..

[66] Pasin T., Benassi V., Moreira E., Jorge J., Polizeli M. (2014). Prospecting filamentous fungi for amylase production: Standardization of Aspergillus japonicus culture conditions. Biotechnol. J. Int..

[67] Souza L.T.A., Oliveira J.S., dos Santos V.L., Regis W.C., Santoro M.M., Resende R.R. (2014). Lipolytic potential of Aspergillus japonicus LAB01: Production, partial purification, and characterisation of an extracellular lipase. BioMed Res. Int..

[68] Karanam S.K., Medicherla N.R. (2008). Enhanced lipase production by mutation induced Aspergillus japonicus. Afr. J. Biotechnol..

[69] Niyonzima F.N., Veena S., More S.S. (2020). Industrial Production and Optimization of Microbial Enzymes. Microbial Enzymes: Roles and Applications in Industries.

[70] Tamminen A., Kramer A., Labes A., Wiebe M.G. (2014). Production of scopularide A in submerged culture with Scopulariopsis brevicaulis. Microb. Cell Factories.

[71] Macchione M.M., Merheb C.W., Gomes E., Da Silva R. (2008). Protease production by different thermophilic fungi. Appl. Biochem. Biotechnol..

[72] Larcher G., Bouchara J.-P., Annaix V., Symoens F., Chabasse D., Tronchin G. (1992). Purification and characterization of a fibrinogenolytic serine proteinase from Aspergillus fumigatus culture filtrate. FEBS Lett..

[73] Farnell E., Rousseau K., Thornton D.J., Bowyer P., Herrick S.E. (2012). Expression and secretion of Aspergillus fumigatus proteases are regulated in response to different protein substrates. Fungal Biol..

[74] Kumar S., Sharma N.S., Saharan M.R., Singh R. (2005). Extracellular acid protease from Rhizopus oryzae: Purification and characterization. Process Biochem..

[75] Benabda O., M’hir S., Kasmi M., Mnif W., Hamdi M. (2019). Optimization of protease and amylase production by Rhizopus oryzae cultivated on bread waste using solid-state fermentation. J. Chem..

[76] Abraham L.D., Breuil C. (1996). Isolation and characterization of a subtilisin-like serine proteinase secreted by the sap-staining fungus Ophiostoma piceae. Enzym. Microb. Technol..

[77] de Oliveira T.B., Gomes E., Rodrigues A. (2015). Thermophilic fungi in the new age of fungal taxonomy. Extremophiles.

[78] Zucconi L., Pagano S., Fenice M., Selbmann L., Tosi S., Onofri S. (1996). Growth temperature preferences of fungal strains from Victoria Land, Antarctica. Polar Biol..

[79] Lasa I., Berenguer J. (1993). Thermophilic enzymes and their biotechnological potential. Microbiologia.

[80] Mikhailova A.G., Khairullin R.F., Demidyuk I.V., Kostrov S.V., Grinberg N.V., Burova T.V., Grinberg V.Y., Rumsh L.D. (2014). Cloning, sequencing, expression, and characterization of thermostability of oligopeptidase B from Serratia proteamaculans, a novel psychrophilic protease. Protein Expr. Purif..

[81] Wancura J.H., Rosset D.V., Tres M.V., Oliveira J.V., Mazutti M.A., Jahn S.L. (2018). Production of biodiesel catalyzed by lipase from Thermomyces lanuginosus in its soluble form. Can. J. Chem. Eng..

[82] Pathak A.P., Rathod M.G. (2018). A Review on Alkaline Protease Producers and Their Biotechnological Perspectives.

[83] Huynh H.H., Arioka M. (2016). Functional expression and characterization of a glucuronoyl esterase from the fungus Neurospora crassa: Identification of novel consensus sequences containing the catalytic triad. J. Gen. Appl. Microbiol..

[84] Singh B. (2016). Myceliophthora thermophila syn. Sporotrichum thermophile: A thermophilic mould of biotechnological potential. Crit. Rev. Biotechnol..

[85] de Oliveira T.B., Rodrigues A. (2019). Ecology of Thermophilic Fungi. Fungi in Extreme Environments: Ecological Role and Biotechnological Significance.

[86] Singh B., Satyanarayana T. (2008). Phytase production by a thermophilic mould Sporotrichum thermophile in solid state fermentation and its potential applications. Bioresour. Technol..

[87] Dilokpimol A., Mäkelä M.R., Cerullo G., Zhou M., Varriale S., Gidijala L., Brás J.L., Jütten P., Piechot A., Verhaert R. (2018). Fungal glucuronoyl esterases: Genome mining based enzyme discovery and biochemical characterization. New Biotechnol..

[88] Salwan R., Sharma V. (2019). Proteases from Extremophilic Fungi: A Tool for White Biotechnology. Recent Advancement in White Biotechnology Through Fungi.

[89] Kieliszek M., Kot A.M., Bzducha-Wróbel A., BŁażejak S., Gientka I., Kurcz A. (2017). Biotechnological use of Candida yeasts in the food industry: A review. Fungal Biol. Rev..

[90] Naveed M., Nadeem F., Mehmood T., Bilal M., Anwar Z., Amjad F. (2021). Protease—a versatile and ecofriendly biocatalyst with multi-industrial applications: An updated review. Catal. Lett..

[91] El-Khonezy M.I., Elgammal E.W., Ahmed E.F., Abd-Elaziz A.M. (2021). Detergent stable thiol-dependant alkaline protease produced from the endophytic fungus Aspergillus ochraceus BT21: Purification and kinetics. Biocatal. Agric. Biotechnol..

[92] Niyonzima F.N., More S.S. (2013). Screening and optimization of cultural parameters for an alkaline protease production by Aspergillus terreus gr. under submerged fermentation. Int. J. Pharm. Bio. Sci..

[93] Yadav V.K., Singh V., Mishra V. (2019). Alkaline Protease: A Tool to Manage Solid Waste and Its Utility in Detergent Industry. Microbial Genomics in Sustainable Agroecosystems.

[94] Martinelli P., Rugarli E.I. (2010). Emerging roles of mitochondrial proteases in neurodegeneration. Biochim. Et Biophys. Acta (BBA)-Bioenerg..

[95] Hariharan A., Rajadurai U.M., Palanivel I. (2019). Isolation, Purification and Mass Production of Protease from Bacillus subtilis. https://ssrn.com/abstract=3370124.

[96] Devi M.K., Banu A.R., Gnanaprabhal G., Pradeep B., Palaniswamy M. (2008). Purification, characterization of alkaline protease enzyme from native isolate Aspergillus niger and its compatibility with commercial detergents. Indian J. Sci. Technol..

[97] Shahid T., Muhammad S., Ahmed K. (2016). Enzyme Proteases Used in Laundry Detergents Engineering a Review. Sci. Int..

[98] Corvo I., Ferraro F., Merlino A., Zuberbühler K., O’Donoghue A.J., Pastro L., Pi-Denis N., Basika T., Roche L., McKerrow J.H. (2018). Substrate specificity of cysteine proteases beyond the S2 Pocket: Mutagenesis and molecular dynamics investigation of Fasciola hepatica Cathepsins L.. Front. Mol. Biosci..

[99] Ou J.-F., Zhu M.-J. (2012). An overview of current and novel approaches for microbial neutral protease improvement. Int. J. Mod. Biol. Med.

[100] Theron L.W., Divol B. (2014). Microbial aspartic proteases: Current and potential applications in industry. Appl. Microbiol. Biotechnol..

[101] Dunn B.M. (2010). Introduction to the aspartic proteinase family. Aspartic Acid Proteases Ther. Targets.

[102] Jiang L., Zhang X., Zhou Y., Chen Y., Luo Z., Li J., Yuan C., Huang M. (2018). Halogen bonding for the design of inhibitors by targeting the S1 pocket of serine proteases. RSC Adv..

[103] Jiang L., Yuan C., Huang M. (2021). A general strategy to inhibit serine protease by targeting its autolysis loop. FASEB J..

[104] Rawlings N.D., Barrett A.J. (1995). [13] Evolutionary families of metallopeptidases. Methods Enzymol..

[105] Cheng M., Takenaka S., Aoki S., Murakami S., Aoki K. (2009). Purification and characterization of an eggshell membrane decomposing protease from Pseudomonas aeruginosa strain ME-4. J. Biosci. Bioeng..

[106] Sims A.H., Dunn-Coleman N.S., Robson G.D., Oliver S.G. (2004). Glutamic protease distribution is limited to filamentous fungi. FEMS Microbiol. Lett..

[107] Kuan C.S., Ng K.P., Yew S.M., Meleh H.U., Seow H.F., How K.N., Yeo S.K., Jee J.M., Tan Y.-C., Yee W.-Y. (2020). Comparative Genomic and Phenotypic Analyses of Pathogenic Fungi Neoscytalidium Dimidiatum and Bipolaris Papendorfii, Isolated From Human Skin Scraping. Res. Sq..

[108] Mamo J., Assefa F. (2018). The role of microbial aspartic protease enzyme in food and beverage industries. J. Food Qual..

[109] Razzaq A., Shamsi S., Ali A., Ali Q., Sajjad M., Malik A., Ashraf M. (2019). Microbial proteases applications. Front. Bioeng. Biotechnol..

[110] Erjavec J., Kos J., Ravnikar M., Dreo T., Sabotič J. (2012). Proteins of higher fungi–from forest to application. Trends Biotechnol..

[111] Shaba A., Baba J. (2012). Screening of Pleurotus ostreatus and Gleophylum sepiarium strains for extracellular protease enzyme production. Bayero J. Pure Appl. Sci..

[112] Papagianni M. (2004). Fungal morphology and metabolite production in submerged mycelial processes. Biotechnol. Adv..

[113] Wang H., Ng T. (2001). Pleureryn, a novel protease from fresh fruiting bodies of the edible mushroom Pleurotus eryngii. Biochem. Biophys. Res. Commun..

[114] Musatti A., Ficara E., Mapelli C., Sambusiti C., Rollini M. (2017). Use of solid digestate for lignocellulolytic enzymes production through submerged fungal fermentation. J. Environ. Manag..

[115] Burdsall H.H., Volk T.J. (2008). Armillaria solidipes, an older name for the fungus called Armillaria ostoyae. North Am. Fungi.

[116] Faraco V., Palmieri G., Festa G., Monti M., Sannia G., Giardina P. (2005). A new subfamily of fungal subtilases: Structural and functional analysis of a Pleurotus ostreatus member. Microbiology.

[117] Nurika I., Suhartini S., Barker G.C. (2020). Biotransformation of tropical lignocellulosic feedstock using the brown rot fungus Serpula lacrymans. Waste Biomass Valorization.

[118] Cha W.-S., Park S.-S., Kim S.-J., Choi D. (2010). Biochemical and enzymatic properties of a fibrinolytic enzyme from Pleurotus eryngii cultivated under solid-state conditions using corn cob. Bioresour. Technol..

[119] Ng T.B., Wong J.H., Cheung R.C.F., Tse T.F., Tam T., Chan H. (2014). Mushrooms: Proteins, polysaccharidepeptide complexes and polysaccharides with antiproliferative and anticancer activities. Int. J. Cancer Res. Prev..

[120] Lv H., Kong Y., Yao Q., Zhang B., Leng F.-w., Bian H.-j., Balzarini J., Van Damme E., Bao J.-k. (2009). Nebrodeolysin, a novel hemolytic protein from mushroom Pleurotus nebrodensis with apoptosis-inducing and anti-HIV-1 effects. Phytomedicine.

[121] Berne S., Križaj I., Pohleven F., Turk T., Maček P., Sepčić K. (2002). Pleurotus and Agrocybe hemolysins, new proteins hypothetically involved in fungal fruiting. Biochim. Et Biophys. Acta (BBA) Gen. Subj..

[122] Sumantha A., Larroche C., Pandey A. (2006). Microbiology and industrial biotechnology of food-grade proteases: A perspective. Food Technol. Biotechnol..

[123] Sandhya C., Nampoothiri K.M., Pandey A. (2005). Microbial Proteases. Microbial Enzymes and Biotransformations.

[124] dos Santos Aguilar J.G., Sato H.H. (2018). Microbial proteases: Production and application in obtaining protein hydrolysates. Food Res. Int..

[125] Baird T.T., Wright W.D., Craik C.S. (2006). Conversion of trypsin to a functional threonine protease. Protein Sci..

[126] Jashni M.K., Dols I.H., Iida Y., Boeren S., Beenen H.G., Mehrabi R., Collemare J., de Wit P.J. (2015). Synergistic action of a metalloprotease and a serine protease from Fusarium oxysporum f. sp. lycopersici cleaves chitin-binding tomato chitinases, reduces their antifungal activity, and enhances fungal virulence. Mol. Plant-Microbe Interact..

[127] Barrett A.J. (1994). [1] Classification of peptidases. Methods Enzymol..

[128] Brocklehurst K., Philpott M.P. (2013). Cysteine proteases: Mode of action and role in epidermal differentiation. Cell Tissue Res..

[129] Yegin S., Fernandez-Lahore M., Salgado A.J.G., Guvenc U., Goksungur Y., Tari C. (2011). Aspartic proteinases from Mucor spp. in cheese manufacturing. Appl. Microbiol. Biotechnol..

[130] Da Silva R.R., Souto T.B., de Oliveira T.B., de Oliveira L.C.G., Karcher D., Juliano M.A., Juliano L., de Oliveira A.H., Rodrigues A., Rosa J.C. (2016). Evaluation of the catalytic specificity, biochemical properties, and milk clotting abilities of an aspartic peptidase from Rhizomucor miehei. J. Ind. Microbiol. Biotechnol..

[131] Dietrich F.S., Voegeli S., Brachat S., Lerch A., Gates K., Steiner S., Mohr C., Pöhlmann R., Luedi P., Choi S. (2004). The Ashbya gossypii genome as a tool for mapping the ancient Saccharomyces cerevisiae genome. Science.

[132] Feldmann H. (2000). Génolevures—A Novel Approach to ‘Evolutionary Genomics’.

[133] Kellis M., Birren B.W., Lander E.S. (2004). Proof and evolutionary analysis of ancient genome duplication in the yeast Saccharomyces cerevisiae. Nature.

[134] Neto Y.A.A.H., de Souza Motta C.M., Cabral H. (2013). Optimization of metalloprotease production by Eupenicillium javanicum in both solid state and submerged bioprocesses. Afr. J. Biochem. Res..

[135] Erez E., Fass D., Bibi E. (2009). How intramembrane proteases bury hydrolytic reactions in the membrane. Nature.

[136] Shafee T. (2014). Evolvability of a Viral Protease: Experimental Evolution of Catalysis, Robustness and Specificity.

[137] Madhavan A., Arun K., Binod P., Sirohi R., Tarafdar A., Reshmy R., Awasthi M.K., Sindhu R. (2021). Design of novel enzyme biocatalysts for industrial bioprocess: Harnessing the power of protein engineering, high throughput screening and synthetic biology. Bioresour. Technol..

[138] Veloorvalappil N.J., Robinson B.S., Selvanesan P., Sasidharan S., Kizhakkepawothail N.U., Sreedharan S., Prakasan P., Moolakkariyil S.J., Sailas B. (2013). Versatility of microbial proteases. Adv. Enzym. Res..

[139] Shankar R., Upadhyay P.K., Kumar M. (2021). Protease Enzymes: Highlights on Potential of Proteases as Therapeutics Agents. Int. J. Pept. Res. Ther..

[140] Chapman J., Ismail A.E., Dinu C.Z. (2018). Industrial applications of enzymes: Recent advances, techniques, and outlooks. Catalysts.

[141] Timson D.J. (2019). Four challenges for better biocatalysts. Fermentation.

[142] Banerjee G., Ray A.K. (2017). Impact of microbial proteases on biotechnological industries. Biotechnol. Genet. Eng. Rev..

[143] Ademosun M.T., Omoba O.S., Olagunju A.I. (2021). Antioxidant properties, glycemic indices, and carbohydrate hydrolyzing enzymes activities of formulated ginger-based fruit drinks. J. Food Biochem..

[144] Kumar D., Bhalla T.C. (2005). Microbial proteases in peptide synthesis: Approaches and applications. Appl. Microbiol. Biotechnol..

[145] Ni X., Yue L., Chi Z., Li J., Wang X., Madzak C. (2009). Alkaline protease gene cloning from the marine yeast Aureobasidium pullulans HN2-3 and the protease surface display on Yarrowia lipolytica for bioactive peptide production. Mar. Biotechnol..

[146] Kanda S., Aimi T., Kano S., Ishihara S., Kitamoto Y., Morinaga T. (2008). Ambient pH signaling regulates expression of the serine protease gene (spr1) in pine wilt nematode-trapping fungus, Monacrosporium megalosporum. Microbiol. Res..

[147] Tzean Y., Chou T.-H., Hsiao C.-C., Shu P.-Y., Walton J.D., Tzean S.-S. (2016). Cloning and characterization of cuticle-degrading serine protease from nematode-trapping fungus Arthrobotrys musiformis. Mycoscience.

[148] Yang J., Zhang K.-Q. (2014). Biological Control of Plant-Parasitic Nematodes by Nematophagous Fungi. Nematode-Trapping Fungi.

[149] Ao X.-L., Yu X., Wu D.-T., Li C., Zhang T., Liu S.-L., Chen S.-J., He L., Zhou K., Zou L.-K. (2018). Purification and characterization of neutral protease from Aspergillus oryzae Y1 isolated from naturally fermented broad beans. AMB Express.

[150] Benmrad M.O., Mechri S., Jaouadi N.Z., Elhoul M.B., Rekik H., Sayadi S., Bejar S., Kechaou N., Jaouadi B. (2019). Purification and biochemical characterization of a novel thermostable protease from the oyster mushroom Pleurotus sajor-caju strain CTM10057 with industrial interest. BMC Biotechnol..

[151] Illuri R., Kumar M., Eyini M., Veeramanikandan V., Almaary K.S., Elbadawi Y.B., Biraqdar M., Balaji P. (2021). Production, partial purification and characterization of ligninolytic enzymes from selected basidiomycetes mushroom fungi. Saudi J. Biol. Sci..

[152] Jaouadi B., Jaouadi N.Z., Rekik H., Naili B., Beji A., Dhouib A., Bejar S. (2013). Biochemical and molecular characterization of Pseudomonas aeruginosa CTM50182 organic solvent-stable elastase. Int. J. Biol. Macromol..

[153] Mandujano-González V., Villa-Tanaca L., Anducho-Reyes M.A., Mercado-Flores Y. (2016). Secreted fungal aspartic proteases: A review. Rev. Iberoam. De Micol..

[154] Rantasalo A., Vitikainen M., Paasikallio T., Jäntti J., Landowski C.P., Mojzita D. (2019). Novel genetic tools that enable highly pure protein production in Trichoderma reesei. Sci. Rep..

[155] Jiang C., Lv G., Tu Y., Cheng X., Duan Y., Zeng B., He B. (2021). Applications of CRISPR/Cas9 in the Synthesis of Secondary Metabolites in Filamentous Fungi. Front. Microbiol..

[156] Liu Q., Gao R., Li J., Lin L., Zhao J., Sun W., Tian C. (2017). Development of a genome-editing CRISPR/Cas9 system in thermophilic fungal Myceliophthora species and its application to hyper-cellulase production strain engineering. Biotechnol. Biofuels.

[157] Adalsteinsson B.T., Kristjansdottir T., Merre W., Helleux A., Dusaucy J., Tourigny M., Fridjonsson O., Hreggvidsson G.O. (2021). Efficient genome editing of an extreme thermophile, Thermus thermophilus, using a thermostable Cas9 variant. Sci. Rep..

[158] Katayama T., Tanaka Y., Okabe T., Nakamura H., Fujii W., Kitamoto K., Maruyama J.-i. (2016). Development of a genome editing technique using the CRISPR/Cas9 system in the industrial filamentous fungus Aspergillus oryzae. Biotechnol. Lett..

[159] Jin F.-J., Hu S., Wang B.-T., Jin L. (2021). Advances in genetic engineering technology and its application in the industrial fungus Aspergillus oryzae. Front. Microbiol..

[160] Zou Z., Liu F., Selin C., Fernando W. (2020). Generation and characterization of a virulent Leptosphaeria maculans isolate carrying a mutated AvrLm7 gene using the CRISPR/Cas9 system. Front. Microbiol..

[161] Chen B.-X., Wei T., Ye Z.-W., Yun F., Kang L.-Z., Tang H.-B., Guo L.-Q., Lin J.-F. (2018). Efficient CRISPR-Cas9 gene disruption system in edible-medicinal mushroom Cordyceps militaris. Front. Microbiol..

[162] Das S.K., Masuda M., Sakurai A., Sakakibara M. (2010). Medicinal uses of the mushroom Cordyceps militaris: Current state and prospects. Fitoterapia.

[163] Gardiner D.M., Kazan K. (2018). Selection is required for efficient Cas9-mediated genome editing in Fusarium graminearum. Fungal Biol..

[164] Joshi R. (2018). A review of Fusarium oxysporum on its plant interaction and industrial use. J. Med. Plants Stud..

[165] Schuster M., Schweizer G., Kahmann R. (2018). Comparative analyses of secreted proteins in plant pathogenic smut fungi and related basidiomycetes. Fungal Genet. Biol..

[166] Olicón-Hernández D.R., Araiza-Villanueva M.G., Pardo J.P., Aranda E., Guerra-Sánchez G. (2019). New insights of Ustilago maydis as yeast model for genetic and biotechnological research: A review. Curr. Microbiol..

[167] Matsu-Ura T., Baek M., Kwon J., Hong C. (2015). Efficient gene editing in Neurospora crassa with CRISPR technology. Fungal Biol. Biotechnol..

[168] Qin H., Xiao H., Zou G., Zhou Z., Zhong J.-J. (2017). CRISPR-Cas9 assisted gene disruption in the higher fungus Ganoderma species. Process Biochem..

[169] Yang H., Wu T., Zhang K. (2003). Effects of Extracts of Chinese Medicines on Ganoderma lucidum in Submerged Culture. Acta Microbiol. Sin..

[170] Schuster M., Schweizer G., Reissmann S., Kahmann R. (2016). Genome editing in Ustilago maydis using the CRISPR–Cas system. Fungal Genet. Biol..

[171] Al Abdallah Q., Souza A.C.O., Martin-Vicente A., Ge W., Fortwendel J.R. (2018). Whole-genome sequencing reveals highly specific gene targeting by in vitro assembled Cas9-ribonucleoprotein complexes in Aspergillus fumigatus. Fungal Biol. Biotechnol..

[172] Zhang Q., Xing H.-L., Wang Z.-P., Zhang H.-Y., Yang F., Wang X.-C., Chen Q.-J. (2018). Potential high-frequency off-target mutagenesis induced by CRISPR/Cas9 in Arabidopsis and its prevention. Plant Mol. Biol..

[173] Doench J.G., Fusi N., Sullender M., Hegde M., Vaimberg E.W., Donovan K.F., Smith I., Tothova Z., Wilen C., Orchard R. (2016). Optimized sgRNA design to maximize activity and minimize off-target effects of CRISPR-Cas9. Nat. Biotechnol..

[174] Song L., Ouedraogo J.-P., Kolbusz M., Nguyen T.T.M., Tsang A. (2018). Efficient genome editing using tRNA promoter-driven CRISPR/Cas9 gRNA in Aspergillus niger. PLoS ONE.

[175] Huang L., Dong H., Zheng J., Wang B., Pan L. (2019). Highly efficient single base editing in Aspergillus niger with CRISPR/Cas9 cytidine deaminase fusion. Microbiol. Res..

[176] Sharma K.M., Kumar R., Panwar S., Kumar A. (2017). Microbial alkaline proteases: Optimization of production parameters and their properties. J. Genet. Eng. Biotechnol..

[177] Ward O.P. (2012). Production of recombinant proteins by filamentous fungi. Biotechnol. Adv..

[178] Ravanelli S., den Brave F., Hoppe T. (2020). Mitochondrial quality control governed by ubiquitin. Front. Cell Dev. Biol..

[179] Jadiya P., Tomar D. (2020). Mitochondrial protein quality control mechanisms. Genes.

[180] Deshwal S., Fiedler K.U., Langer T. (2020). Mitochondrial proteases: Multifaceted regulators of mitochondrial plasticity. Annu. Rev. Biochem..

[181] Quiros P.M., Langer T., Lopez-Otin C. (2015). New roles for mitochondrial proteases in health, ageing and disease. Nat. Rev. Mol. Cell Biol..

[182] Hofsetz E., Huesgen P.F., Trifunovic A. (2021). Identification of Putative Mitochondrial Protease Substrates. Mitochondrial Gene Expression.

[183] Voos W. (2013). Chaperone–protease networks in mitochondrial protein homeostasis. Biochim. Et Biophys. Acta (BBA) Mol. Cell Res..

[184] Teixeira P.F., Glaser E. (2013). Processing peptidases in mitochondria and chloroplasts. Biochim. Et Biophys. Acta (BBA) Mol. Cell Res..

[185] Weiss-Sadan T., Gotsman I., Blum G. (2017). Cysteine proteases in atherosclerosis. FEBS J..

[186] Liu J., Sukhova G.K., Yang J.-T., Sun J., Ma L., Ren A., Xu W.-H., Fu H., Dolganov G.M., Hu C. (2006). Cathepsin L expression and regulation in human abdominal aortic aneurysm, atherosclerosis, and vascular cells. Atherosclerosis.

[187] Jaffer F.A., Vinegoni C., John M.C., Aikawa E., Gold H.K., Finn A.V., Ntziachristos V., Libby P., Weissleder R. (2008). Real-time catheter molecular sensing of inflammation in proteolytically active atherosclerosis. Circulation.

[188] Weitoft T., Larsson A., Manivel V.A., Lysholm J., Knight A., Rönnelid J. (2015). Cathepsin S and cathepsin L in serum and synovial fluid in rheumatoid arthritis with and without autoantibodies. Rheumatology.

[189] Appelqvist H., Wäster P., Kågedal K., Öllinger K. (2013). The lysosome: From waste bag to potential therapeutic target. J. Mol. Cell Biol..

[190] Wu D., Wei Y., Bleier B.S. (2018). Emerging role of proteases in the pathogenesis of chronic rhinosinusitis with nasal polyps. Front. Cell. Infect. Microbiol..

[191] Stentzel S., Teufelberger A., Nordengrün M., Kolata J., Schmidt F., Van Crombruggen K., Michalik S., Kumpfmüller J., Tischer S., Schweder T. (2017). Staphylococcal serine protease–like proteins are pacemakers of allergic airway reactions to Staphylococcus aureus. J. Allergy Clin. Immunol..

[192] Kale S.L., Agrawal K., Gaur S.N., Arora N. (2017). Cockroach protease allergen induces allergic airway inflammation via epithelial cell activation. Sci. Rep..

[193] Teufelberger A.R., Nordengrün M., Braun H., Maes T., De Grove K., Holtappels G., O’Brien C., Provoost S., Hammad H., Gonçalves A. (2018). The IL-33/ST2 axis is crucial in type 2 airway responses induced by Staphylococcus aureus–derived serine protease–like protein D. J. Allergy Clin. Immunol..

[194] López-Otín C., Matrisian L.M. (2007). Emerging roles of proteases in tumour suppression. Nat. Rev. Cancer.

[195] Kwon Y.T., Ciechanover A. (2017). The ubiquitin code in the ubiquitin-proteasome system and autophagy. Trends Biochem. Sci..

[196] Liu J., Shaik S., Dai X., Wu Q., Zhou X., Wang Z., Wei W. (2015). Targeting the ubiquitin pathway for cancer treatment. Biochim. Et Biophys. Acta (BBA)-Rev. Cancer.

[197] Green P.H., Lebwohl B., Greywoode R. (2015). Celiac disease. J. Allergy Clin. Immunol..

[198] Makharia G.K.D. (2014). Current and emerging therapy for celiac disease. Front. Med..

[199] Caruso J.A., Akli S., Pageon L., Hunt K.K., Keyomarsi K. (2015). The serine protease inhibitor elafin maintains normal growth control by opposing the mitogenic effects of neutrophil elastase. Oncogene.

[200] Galipeau H.J., Wiepjes M., Motta J.-P., Schulz J.D., Jury J., Natividad J.M., Pinto-Sanchez I., Sinclair D., Rousset P., Martin-Rosique R. (2014). Novel role of the serine protease inhibitor elafin in gluten-related disorders. Am. J. Gastroenterol..

[201] Ghetti B., Tagliavini F., Kovacs G.G., Piccardo P. (2011). 37 Gerstmann–Str ä ussler–Scheinker Disease. Neurodegeneration: The Molecular Pathology of Dementia and Movement Disorders.

[202] Iwasaki Y. (2017). Creutzfeldt-Jakob disease. Neuropathology.

[203] Yoshioka M., Miwa T., Horii H., Takata M., Yokoyama T., Nishizawa K., Watanabe M., Shinagawa M., Murayama Y. (2007). Characterization of a proteolytic enzyme derived from a Bacillus strain that effectively degrades prion protein. J. Appl. Microbiol..

[204] Rajput R., Gupta R. (2013). Thermostable keratinase from Bacillus pumilus KS12: Production, chitin crosslinking and degradation of Sup35NM aggregates. Bioresour. Technol..

[205] Chauhan B., Gupta R. (2004). Application of statistical experimental design for optimization of alkaline protease production from Bacillus sp. RGR-14. Process Biochem..

[206] Reddy C.C., Khilji I.A., Gupta A., Bhuyar P., Mahmood S., AL-Japairai K.A.S., Chua G.K. (2021). Valorization of keratin waste biomass and its potential applications. J. Water Process Eng..

[207] Matkawala F., Nighojkar S., Kumar A., Nighojkar A. (2021). Microbial alkaline serine proteases: Production, properties and applications. World J. Microbiol. Biotechnol..

[208] Chilakamarry C.R., Mahmood S., Saffe S.N.B.M., Arifin M.A.B., Gupta A., Sikkandar M.Y., Begum S.S., Narasaiah B. (2021). Extraction and application of keratin from natural resources: A review. 3 Biotech.

[209] Qiu J., Wilkens C., Barrett K., Meyer A.S. (2020). Microbial enzymes catalyzing keratin degradation: Classification, structure, function. Biotechnol. Adv..

[210] Bhandari S., Poudel D.K., Marahatha R., Dawadi S., Khadayat K., Phuyal S., Shrestha S., Gaire S., Basnet K., Khadka U. (2021). Microbial Enzymes Used in Bioremediation. J. Chem..

[211] Saranya P., Selvi P., Sekaran G. (2019). Integrated thermophilic enzyme-immobilized reactor and high-rate biological reactors for treatment of palm oil-containing wastewater without sludge production. Bioprocess Biosyst. Eng..

[212] Gradisar H., Friedrich J., Krizaj I., Jerala R. (2005). Similarities and specificities of fungal keratinolytic proteases: Comparison of keratinases of Paecilomyces marquandii and Doratomyces microsporus to some known proteases. Appl. Environ. Microbiol..

[213] Espersen R., Huang Y., Falco F.C., Hägglund P., Gernaey K.V., Lange L., Svensson B. (2021). Exceptionally rich keratinolytic enzyme profile found in the rare actinomycetes Amycolatopsis keratiniphila D2T. Appl. Microbiol. Biotechnol..

[214] Barzkar N., Sohail M., Jahromi S.T., Nahavandi R., Khodadadi M. (2021). Marine microbial L-glutaminase: From pharmaceutical to food industry. Appl. Microbiol. Biotechnol..

[215] Bond J.S. (2019). Proteases: History, discovery, and roles in health and disease. J. Biol. Chem..

[216] Rudzińska M., Daglioglu C., Savvateeva L.V., Kaci F.N., Antoine R., Zamyatnin A.A. (2021). Current Status and Perspectives of Protease Inhibitors and Their Combination with Nanosized Drug Delivery Systems for Targeted Cancer Therapy. Drug Des. Dev. Ther..

[217] Awad M.F., El-Shenawy F.S., El-Gendy M.M.A.A., El-Bondkly E.A.M. (2021). Purification, characterization, and anticancer and antioxidant activities of l-glutaminase from Aspergillus versicolor Faesay4. Int. Microbiol..

[218] Qamar S.A., Asgher M., Bilal M. (2020). Immobilization of alkaline protease from Bacillus brevis using Ca-alginate entrapment strategy for improved catalytic stability, silver recovery, and dehairing potentialities. Catal. Lett..

[219] Bholay A., More S., Patil V., Niranjan P. (2012). Bacterial extracellular alkaline proteases and its industrial applications. Int. Res. J. Biol. Sci..

[220] Abad P., Gouzy J., Aury J.-M., Castagnone-Sereno P., Danchin E.G., Deleury E., Perfus-Barbeoch L., Anthouard V., Artiguenave F., Blok V.C. (2008). Genome sequence of the metazoan plant-parasitic nematode Meloidogyne incognita. Nat. Biotechnol..

[221] Nakpathom M., Somboon B., Narumol N. (2009). Papain enzymatic degumming of Thai Bombyx mori silk fibers. J. Microsc. Soc. Thail..

[222] Miao Y., Chen G., Xi X., Ma C., Wang L., Burrows J.F., Duan J., Zhou M., Chen T. (2019). Discovery and rational design of a novel bowman-birk related protease inhibitor. Biomolecules.

[223] Zhai P.-b., Qing J., Li B., Zhang L.-q., Ma L., Chen L. (2018). GP205, a new hepatitis C virus NS3/4A protease inhibitor, displays higher metabolic stability in vitro and drug exposure in vivo. Acta Pharmacol. Sin..

[224] Goris T., Pérez-Valero Á., Martínez I., Yi D., Fernández-Calleja L., San Leon D., Bornscheuer U.T., Magadán-Corpas P., Lombo F., Nogales J. (2021). Repositioning microbial biotechnology against COVID-19: The case of microbial production of flavonoids. Microb. Biotechnol..

[225] Cotabarren J., Lufrano D., Parisi M.G., Obregón W.D. (2020). Biotechnological, biomedical, and agronomical applications of plant protease inhibitors with high stability: A systematic review. Plant Sci..

[226] Rawlings N.D. (2010). Peptidase inhibitors in the MEROPS database. Biochimie.

[227] Garg H., Suri P., Gupta J.C., Talwar G., Dubey S. (2016). Survivin: A unique target for tumor therapy. Cancer Cell Int..

[228] Rawlings N.D., Barrett A.J., Finn R. (2016). Twenty years of the MEROPS database of proteolytic enzymes, their substrates and inhibitors. Nucleic Acids Res..

[229] Gutierrez-Gongora D., Geddes-McAlister J. (2021). From Naturally-Sourced Protease Inhibitors to New Treatments for Fungal Infections. J. Fungi.

[230] Kantyka T., Rawlings N.D., Potempa J. (2010). Prokaryote-derived protein inhibitors of peptidases: A sketchy occurrence and mostly unknown function. Biochimie.

[231] Greenbaum L.M., Sutherland J.H. (1983). Host cathepsin D response to tumor in the normal and pepstatin-treated mouse. Cancer Res..

[232] Kalchev K., Rabadjiev Y., Ganchev D., Tsenova M., Iliev I., Ivanova I. (2013). Study of proteases and protease inhibitors from Streptomyces strains. Bulg. J. Agric. Sci..

[233] Manojlovic N.T., Solujic S., Sukdolak S., Milosev M. (2005). Antifungal activity of Rubia tinctorum, Rhamnus frangula and Caloplaca cerina. Fitoterapia.

[234] Menéndez-Arias L., Tözsér J. (2008). HIV-1 protease inhibitors: Effects on HIV-2 replication and resistance. Trends Pharmacol. Sci..

[235] Chandwani A., Shuter J. (2008). Lopinavir/ritonavir in the treatment of HIV-1 infection: A review. Ther. Clin. Risk Manag..

[236] Fear G., Komarnytsky S., Raskin I. (2007). Protease inhibitors and their peptidomimetic derivatives as potential drugs. Pharmacol. Ther..

[237] Purohit R., Rajendran V., Sethumadhavan R. (2011). Studies on adaptability of binding residues flap region of TMC-114 resistance HIV-1 protease mutants. J. Biomol. Struct. Dyn..

[238] van Maarseveen N.M., Andersson D., Lepšík M., Fun A., Schipper P.J., de Jong D., Boucher C.A., Nijhuis M. (2012). Modulation of HIV-1 Gag NC/p1 cleavage efficiency affects protease inhibitor resistance and viral replicative capacity. Retrovirology.

[239] Liu-Young G., Kozal M.J. (2008). Hepatitis C protease and polymerase inhibitors in development. AIDS Patient Care STDs.

[240] Onoda T., Ono T., Dhar D.K., Yamanoi A., Nagasue N. (2006). Tetracycline analogues (doxycycline and COL-3) induce caspase-dependent and-independent apoptosis in human colon cancer cells. Int. J. Cancer.

[241] Sridhar S.S., Shepherd F.A. (2003). Targeting angiogenesis: A review of angiogenesis inhibitors in the treatment of lung cancer. Lung Cancer.

[242] Rawlings N.D., Tolle D.P., Barrett A.J. (2004). Evolutionary families of peptidase inhibitors. Biochem. J..

[243] Clemente M., Corigliano M.G., Pariani S.A., Sánchez-López E.F., Sander V.A., Ramos-Duarte V.A. (2019). Plant serine protease inhibitors: Biotechnology application in agriculture and molecular farming. Int. J. Mol. Sci..

[244] Peele K.A., Durthi C.P., Srihansa T., Krupanidhi S., Ayyagari V.S., Babu D.J., Indira M., Reddy A.R., Venkateswarulu T. (2020). Molecular docking and dynamic simulations for antiviral compounds against SARS-CoV-2: A computational study. Inform. Med. Unlocked.

[245] Macchiagodena M., Pagliai M., Procacci P. (2020). Identification of potential binders of the main protease 3CLpro of the COVID-19 via structure-based ligand design and molecular modeling. Chem. Phys. Lett..

[246] Rut W., Groborz K., Zhang L., Sun X., Zmudzinski M., Pawlik B., Wang X., Jochmans D., Neyts J., Młynarski W. (2021). SARS-CoV-2 M pro inhibitors and activity-based probes for patient-sample imaging. Nat. Chem. Biol..

[247] Drag M., Salvesen G.S. (2010). Emerging principles in protease-based drug discovery. Nat. Rev. Drug Discov..

[248] Amin S.A., Banerjee S., Ghosh K., Gayen S., Jha T. (2020). Protease targeted COVID-19 drug discovery and its challenges: Insight into viral main protease (Mpro) and papain-like protease (PLpro) inhibitors. Bioorganic Med. Chem..

[249] Lee T.-W., Cherney M.M., Huitema C., Liu J., James K.E., Powers J.C., Eltis L.D., James M.N. (2005). Crystal structures of the main peptidase from the SARS coronavirus inhibited by a substrate-like aza-peptide epoxide. J. Mol. Biol..

[250] Shen S., Wu Y., Liu Y., Wu D. (2017). High drug-loading nanomedicines: Progress, current status, and prospects. Int. J. Nanomed..

[251] Shen S., Du X.-J., Liu J., Sun R., Zhu Y.-H., Wang J. (2015). Delivery of bortezomib with nanoparticles for basal-like triple-negative breast cancer therapy. J. Control. Release.

[252] Park J.E., Park J., Jun Y., Oh Y., Ryoo G., Jeong Y.-S., Gadalla H.H., Min J.S., Jo J.H., Song M.G. (2019). Expanding therapeutic utility of carfilzomib for breast cancer therapy by novel albumin-coated nanocrystal formulation. J. Control. Release.

[253] Gotou T., Shinoda T., Mizuno S., Yamamoto N. (2009). Purification and identification of proteolytic enzymes from Aspergillus oryzae capable of producing the antihypertensive peptide Ile-Pro-Pro. J. Biosci. Bioeng..

[254] Song R., Qiao W., He J., Huang J., Luo Y., Yang T. (2021). Proteases and Their Modulators in Cancer Therapy: Challenges and Opportunities. J. Med. Chem..

[255] Cavaco M., Andreu D., Castanho M.A. (2021). The challenge of peptide proteolytic stability studies: Scarce data, difficult readability, and the need for harmonization. Angew. Chem. Int. Ed..

[256] Verma S., Goand U.K., Husain A., Katekar R.A., Garg R., Gayen J.R. (2021). Challenges of peptide and protein drug delivery by oral route: Current strategies to improve the bioavailability. Drug Dev. Res..

[257] Sharma A., Gupta G., Ahmad T., Mansoor S., Kaur B. (2021). Enzyme engineering: Current trends and future perspectives. Food Rev. Int..

[258] Nyika J.M. (2021). The Use of Microorganism-Derived Enzymes for Bioremediation of Soil Pollutants. Recent Advancements in Bioremediation of Metal Contaminants.

[259] Saravanan A., Kumar P.S., Vo D.-V.N., Jeevanantham S., Karishma S., Yaashikaa P. (2021). A review on catalytic-enzyme degradation of toxic environmental pollutants: Microbial enzymes. J. Hazard. Mater..

[260] Li Q., Yi L., Marek P., Iverson B.L. (2013). Commercial proteases: Present and future. FEBS Lett..

[261] Chen X., Shen H., Shao Y., Ma Q., Niu Y., Shang Z. (2021). A narrative review of proteolytic targeting chimeras (PROTACs): Future perspective for prostate cancer therapy. Transl. Androl. Urol..

